# Exploring of bladder cancer immune-related genes and potential therapeutic targets based on transcriptomic data and Mendelian randomization analysis

**DOI:** 10.3389/fimmu.2025.1607098

**Published:** 2025-07-18

**Authors:** Zhangxiao Xu, Juan Yang, Yira Ma, Bo Tao, Yunpeng He, Jian Wu, Yuan Zhao, Yuanjian Niu, Lijun Wang

**Affiliations:** ^1^ Department of Urinary Surgery, Anning First People’s Hospital Affiliated to Kunming University of Science and Technology, Anning, China; ^2^ Faculty of Life Science and Technology, Kunming University of Science and Technology, Kunming, China

**Keywords:** bladder cancer, Mendelian randomization analysis, WGCNA, CIBERSORT, biomarkers, potential therapeutic targets

## Abstract

**Background:**

Despite advancements in clinical treatment modalities, immune-related molecular mechanisms underlying bladder cancer remain unclear. Therefore, this study aimed to identify immune-related biomarkers and potential therapeutic targets for bladder cancer, thereby contributing to the development of novel therapeutic interventions.

**Methods:**

By integrating data from The Cancer Genome Atlas (TCGA), Gene Expression Omnibus (GEO), and genome-wide association study (GWAS) databases, combined with differential expression analysis, weighted gene co-expression network analysis (WGCNA), and Mendelian randomization analysis, key immune-related genes in bladder cancer were identified. The correlation between these key genes and immune cell infiltration was also analyzed. The diagnostic efficacy of the key genes was evaluated using Receiver Operating Characteristic (ROC) curves and validated using independent public datasets. Finally, Quantitative real-time polymerase chain reaction (qRT-PCR) was performed to confirm the potential value of these molecular markers in bladder cancer.

**Results:**

Differential expression analysis revealed 2,033 bladder cancer-related genes. WGCNA identified 1,391 immune-related genes and Mendelian randomization analysis identified 187 candidate genes with causal relationships. Eight significantly downregulated genes were identified: LIMS2, TP53INP2, IRAK3, STX2, CYP27A1, IL11RA, KCNMB1, and PDLM7. These genes were significantly associated with immune cell infiltration and exhibited good diagnostic efficacy, as demonstrated by ROC curve analysis and validated in independent public datasets. Furthermore, qRT-PCR experiments showed that LIMS2, IRAK3, STX2, IL11RA, KCNMB1, and PDLM7 were significantly downregulated in the tumor group, consistent with the bioinformatic analysis results, suggesting their potential clinical value.

**Conclusion:**

This study identified six immunoregulatory genes that were significantly negatively associated with bladder cancer risk. These genes may serve not only as potential biomarkers for bladder cancer immunity but also contribute to a deeper understanding of the molecular mechanisms of bladder cancer.

## 1 Introduction

Bladder cancer (BCa) is among the ten most common cancers globally, exhibiting high incidence and mortality rates worldwide and imposing a substantial burden on healthcare systems (1, 2). Globally, studies have reported a 5-year prevalence of 1.72 million cases, 573,000 new cases, and 213,000 deaths from bladder cancer (2). Non-muscle invasive bladder cancer (NMIBC; Ta, T1) and carcinoma *in situ* (CIS) generally have a favorable prognosis; however, up to 40% of cases progress to muscle-invasive bladder cancer (MIBC; T2+) depending on the clinical risk group (3). Most bladder cancers are urothelial carcinomas, with approximately 75% of newly diagnosed patients presenting with NMIBC and 25% presenting with MIBC or metastatic disease (4). Approximately 50% of NMIBC cases are low-grade, whereas most MIBC or metastatic tumors are high-grade (5). In recent decades, rare variants of bladder cancer have been increasingly recognized, including micropapillary, plasmacytoid, nested, sarcomatoid, microcystic, neuroendocrine, squamous, and adenocarcinoma subtypes (6). Statistically, men are approximately three–four times more likely to develop bladder cancer than women; however, women often present with a poorer prognosis at later stages (7). Furthermore, owing to the lack of novel therapies and preventative measures, the detrimental impact of bladder cancer on human health significantly surpasses that of other cancers (8).

Early diagnosis and aggressive treatment are crucial to maximize the delay or control of bladder cancer progression. Common treatments for bladder cancer include surgical resection, radiotherapy, and chemotherapy (9, 10). Although these methods can control bladder cancer progression to some extent, they have limitations. In recent years, immunotherapy has emerged as a novel treatment modality, particularly with the widespread use of immune checkpoint inhibitors (ICIs), which not only improve treatment efficacy but also reduce the risk of recurrence and metastasis (11), offering new hope for bladder cancer patients. In-depth research on the molecular mechanisms underlying bladder cancer has propelled targeted therapy to play an increasingly important role in treatment strategies. Compared with traditional chemotherapeutic agents, molecularly targeted drugs exhibit higher specificity, primarily affecting cancer cells, thus minimizing damage to normal tissues (12). However, achieving effective immunotherapy or targeted therapy for bladder cancer requires identification of molecular markers and therapeutic targets. To date, PD-1/PD-L1 inhibitors, the FGFR inhibitor erdafitinib, and the nectin-4-targeted antibody-drug conjugate enfortumab vedotin (Padcev) have been approved for the treatment of bladder cancer (13, 14). Despite significant advancements in cancer biology and treatment, the prognosis of patients with bladder cancer remains unsatisfactory (15). Therefore, a thorough understanding of the molecular characteristics of bladder cancer, particularly the key molecular pathways and genetic mutations that drive tumorigenesis and contribute to drug resistance, is essential for identifying novel therapeutic targets. Developing more effective drugs based on these new targets will bring new therapeutic hope to bladder cancer patients.

## 2 Materials and methods

### 2.1 Experimental design

**Figure 1:**
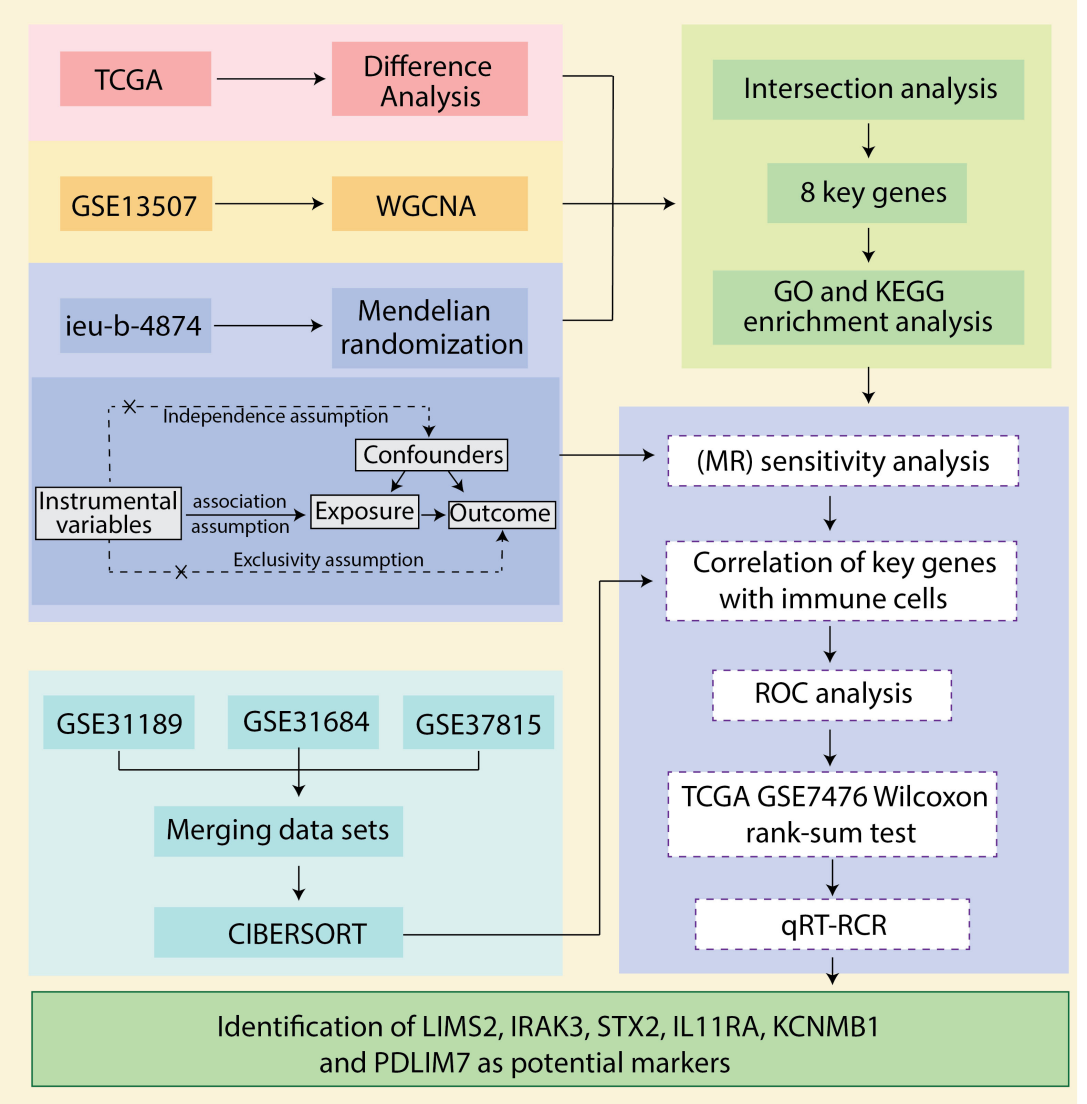
Schematic presentation of the analysis process.

We downloaded bladder cancer-related datasets from The Cancer Genome Atlas (TCGA) database and extracted differentially expressed genes (DEGs). Next, we utilized weighted gene co-expression network analysis (WGCNA) to extract 2033 immune-related genes from the bladder cancer dataset GSE13507. We then employed a two-sample Mendelian randomization (MR) analysis using data from the genome-wide association study (GWAS) database (ebi-a-GCST90018817) to explore the causal relationship between expression quantitative trait loci (eQTLs) and bladder cancer. By overlapping bladder cancer-associated genes, bladder cancer DEGs, and eQTLs that showed significant results in the MR analysis, we identified common genes (CGs) as key genes and prospective biomarkers. Subsequently, we analyzed the correlation between these key genes and immune cell infiltration, assessed the diagnostic efficacy of the key genes using receiver operating characteristic (ROC) curves, and validated the findings using an independent public dataset. Finally, we performed quantitative real-time polymerase chain reaction (qRT-PCR) using clinical samples and *in vitro* cell models to confirm the potential value of these molecular markers in bladder cancer diagnosis and prognosis. The robustness of our findings was further confirmed through a sensitivity analysis. The workflow is illustrated in **Figure 1**.

### 2.2 Data sources and preprocessing

The inclusion criteria for bladder cancer microarray datasets were as follows: (1) each dataset contained at least six samples (with at least three bladder cancer tissue samples and three normal tissue samples), (2) high-quality RNA-seq or microarray data were available, and (3) the dataset did not include any chemically treated or transgenic samples. Based on these criteria, seven independent bladder cancer datasets from the TCGA, Gene Expression Omnibus (GEO), and GWAS databases were integrated. RNA sequencing data and corresponding clinical information for bladder cancer were obtained from TCGA public database. Due to the sufficient sample size and rich clinical information in TCGA, which provides adequate statistical power for detecting differentially expressed genes, TCGA data were used for differential expression analysis. Five microarray datasets were downloaded from the GEO database (GSE13507, GSE31189, GSE31684, GSE37815, and GSE7476). These datasets contain gene expression profiles of bladder cancer and normal tissues. Considering that WGCNA requires a relatively large sample size to improve the statistical power for detecting correlations and is sensitive to outliers, ensuring the completeness and consistency of gene expression data is essential. Therefore, the GSE13507 dataset, which included 196 samples, was used for WGCNA to identify immune-related genes in bladder cancer. For CIBERSORT analysis, the GSE31189, GSE31684, and GSE37815 datasets were standardized to reduce batch effects and then merged, resulting in a dataset containing 163 bladder cancer and 46 normal tissue samples. To ensure the reliability of the study results, the GSE7476 dataset, with no overlapping samples, was used for validation. The eQTL data used in this study were obtained from the IEU Open GWAS database (https://gwas.mrcieu.ac.uk/), from which we collected data from 5,430 eQTL datasets as exposure variables. Additionally, we searched the GWAS database using “bladder cancer” as the keyword and obtained dataset ieu-b-4874 as the outcome data, which included information from 373,295 individuals of European ancestry (1,279 cases and 372,016 controls). As all the data used in this study were publicly available and freely downloadable, no separate ethical approval was required. **Table 1** presents the data sources and the grouping information.

**Table 1:** Data sources and grouping information.

Databases	Datasets	Grouping information		Purpose
		Tumour	Normal	
TCGA	431 samples	412	19	DEGs
GEO	GSE13507	187	9	WGCNA
	GSE31189	52	40	CIBEROST
	GSE31684	93	/	
	GSE37815	18	6	
	GSE7476	9	3	Validation set
GWAS	ebi-GCST90018817	1279	372, 016	MR

### 2.3 Data processing and differential expression gene identification

Following the annotation files, data preprocessing and differential expression gene (DEG) identification began by converting the probe matrix to a gene matrix using a Perl script. Probes associated with multiple genes were excluded and the average number of multiple probes representing the same gene was calculated to determine the final gene expression level. Differential expression analysis between bladder cancer and normal tissues in TCGA dataset was performed using the DESeq2, limma, and Wilcoxon packages. Genes were selected based on an adjusted P-value < 0.05 and an absolute log2 fold change (|log2FC|) greater than 0.585. Heatmaps and volcano plots were generated using the “pheatmap” and “ggplot2” R packages, respectively, to visualize the DEGs (16).

### 2.4 Identification of bladder cancer immune-related genes using WGCNA

A weighted gene co-expression network was constructed using the “WGCNA” package in R (17). First, sample clustering was conducted to screen outlier samples. Next, the gene expression matrix was converted into a similarity matrix by calculating the Pearson’s correlation values between every two genes. Moreover, the similarity matrix was transformed into an adjacency matrix by setting amn = |cmn| β, where cmn represents the Pearson’s correlation values between every two genes, amn is the adjacency between every two genes, and β is a soft threshold that can regulate the correlations among genes. Furthermore, the soft thresholding power was set to five (β = 5, R^2^ = 0.85) based on scale independence and mean connectivity, and the adjacency matrix was transformed into a topological overlap matrix, where gene interactions maximally conformed to the scale-free distribution. Moreover, the modules were identified by hierarchical clustering using dynamic tree-cutting algorithms, using unsigned as the network construction type, and deepSplit was set to two to divide the number of modules and a minimum module size of 30 for the gene dendrogram. Finally, the dissimilarity of the module eigengenes (MES) was calculated for the module dendrogram and some modules (dissimilarity of module eigengenes < 0.3) were merged to obtain the ultimate network.

### 2.5 Instrumental variable selection

To enhance the accuracy and validity of the analysis of the causal relationship between eQTLs and bladder cancer risk, stringent quality control steps were implemented for IV selection: (1) Single nucleotide polymorphisms (SNPs) significantly associated with eQTLs were selected using a P-value threshold of <5×10^−8^ (18); (2) Linkage disequilibrium (LD) was controlled by setting R²= 0.001 and an LD region width of 10,000 kb to maintain SNP independence (19); (3) SNPs directly associated with bladder cancer (P < 5×10^-8^) were excluded (20); (4) The F-statistic for each SNP was calculated using the formula.


$$F=\frac{R^{2}(n-2)}{1-R^{2}}$$

where n is the sample size, R² is the proportion of variance in the exposure explained by the SNP, MAF is the minor allele frequency, and β is the allele effect size. SNPs with weak instrumental variables (F < 10) were excluded (21). (5) A harmonization process aligned effect directions and alleles, ensuring that SNPs had a minor allele frequency (MAF) > 0.01, and removed palindromic and ambiguous SNPs (22).

### 2.6 Mendelian randomization analysis

In this study, the “TwoSampleMR” package in R was used to perform a Mendelian Randomization (MR) analysis between instrumental variables (IVs) and outcome variables. Five different methods—Inverse Variance Weighted (IVW), Weighted Median (WM), MR-Egger, Simple Mode, and Weighted Mode—were employed to evaluate the causal relationship between 5,430 eQTLs and bladder cancer. The IVW method, which combines the Wald estimates of each SNP through meta-analysis and does not assume pleiotropy, was considered the most effective approach for causal inference in this study (23). The MR-Egger method, which is robust against potential horizontal pleiotropy (indicated by an MR-Egger intercept of P< 0.05), provides a robust estimate (24). The WM method derives a median estimate from the distribution of individual SNP effect sizes weighted by their precision and provides reliable causal estimates, particularly when valid IVs account for > 50% of the data (25). The simple model can tolerate pleiotropic effects but may be less efficient than IVW (26). The weighted model adjusts for scenarios where the pleiotropy assumption may be violated (27). Based on the results of the MR analysis, genes were selected according to the following criteria: (1) the IVW method showed a P-value < 0.05; (2) the results of all five analytical methods met the directional consistency criterion [that is, the odds ratios (ORs) were in the same direction]; (3) the IVW results were corrected using the false discovery rate method with an adjusted P-value < 0.05; and (4) pleiotropy analysis indicated no evidence of pleiotropy (P > 0.05).

### 2.7 Sensitivity analysis

A series of sensitivity analyses was performed to validate the robustness of our findings: (1) Cochran’s Q test using IVW and MR-Egger assessed heterogeneity among IVs, and funnel plots revealed significant heterogeneity (P< 0.05) in the selected SNPs (28); (2) horizontal pleiotropy in the MR study was assessed using MR-Egger regression, where an MR-Egger intercept (P< 0.05) indicated substantial horizontal pleiotropy (29); and (3) leave-one-out analysis sequentially excluded one SNP at a time and recalculated the MR estimates from the remaining SNPs to determine the influence of individual SNPs on the collective results (30, 31). These procedures were performed using the “TwoSampleMR” and “MendelianRandomization” R packages in the R software (version 4.3.2).

### 2.8 Gene ontology enrichment analysis of eight genes

To further explore the biological functions of the key genes, Gene Ontology (GO) annotation and Kyoto Encyclopedia of Genes and Genomes (KEGG) pathway enrichment analyses were conducted. Gene ontology (GO) enrichment analysis was performed using GO terms for molecular function (MF), biological process (BP), and cellular component (CC) using the “clusterProfiler,” “org.Hs.eg.db,” and “enrichplot” R packages (32). The results were visualized using the “ggplot2” R package with a significance threshold of P < 0.05.

### 2.9 Immune infiltration analysis

Immune cell infiltration was assessed using the CIBERSORT algorithm (33), implemented in R using the “CIBERSORT” package. This algorithm estimated the proportions of the 22 immune cell types in the dataset by estimating the relative subsets of RNA transcripts. Samples with CIBERSORT scores (P > 0.05) were excluded to ensure the accuracy of the algorithm. The deconvolution algorithm was then applied to the gene expression profiles of the remaining samples for CIBERSORT analysis.

### 2.10 Assessment of diagnostic value of hub genes

To further analyze the ability of the five hub genes to discriminate between tumor and non-tumor samples, receiver operating characteristic (ROC) analysis was performed using the “survivalROC” R package (34) on TCGA data to investigate the diagnostic value of these key genes.

### 2.11 Validation of hub genes in training and validation sets

To further validate the potential value of the eight key genes, their expression levels were compared between bladder tumors and normal tissues in both the TCGA and GSE7476 datasets. Wilcoxon rank-sum test was used to assess the significance of any observed differences.

### 2.12 Quantitative real-time polymerase chain reaction

The human uroepithelial cell line SV-HUC-1 and bladder cancer cell lines 5637, T24, and HT1376 (Yunnan Tengyue Biotech Co. E2112) were cultured in RPMI-1640 medium (Gibco, cat. no. C11875500BT; SV-HUC-1, 5637, HT1376) or McCoy’s 5A medium (EvaCell, cat. no. E2110; T24). All cultures were maintained at 37°C with 5% CO2, supplemented with 10% fetal bovine serum (FBS; Gibco, cat. no. A5256701), and 1% penicillin-streptomycin (Gibco, cat. no. 15140122). Total RNA extraction was performed using either the RNAfast200 kit (Shanghai Feijie Biotechnology Co., Ltd.) or TRIzol-A+ reagent. Reverse transcription was performed using Evo M-MLV Reverse Transcription Reagent Premix (Hunan Acres Bio; cat. no. AG11706) or a commercial reverse transcription kit. Gene expression analysis was performed using the SYBR Green Pro Taq Hs Pre-mixed qRT-PCR Kit (Hunan Acres Bio, cat. no. AG11701), or SYBR Green PCR Master Mix on an Applied Biosystems 7500 real-time PCR system. Relative gene expression levels were calculated using the 2-ΔΔCt method, normalized to GAPDH. The primer sequences used are listed in **Table 2**.

**Table 2:** Primers of hub genes.

Genes	Forword	Reverse
LIMS2	TGTGTGAGCTGCTTCTCCTG	CACCTCTTACACACGGGCTT
TP53INP2	CACCATAGTGCTAGAGCCCG	CAATTCCCCTTCGCTGAGGT
IRAK3	ACCATGCTCGGTCATCTGTG	ATGTTCTAGGTGGGACCGGA
STX2	GAGTGGGAACCGGACTTCAG	GTCTGTGGTGGTTCTCCCAG
CYP27A1	CCAGGATCCAGCACCCATTT	CCACACTCTTCAACTCCCCC
IL11RA	CAGGCAGACAGCACTGATGA	TGGATGGACTCCTCCTCTGG
KCNMB1	TGTGTGCCGTCATCACCTAC	CCTGGTCCCTGATGTTGGTC
PDLIM7	GTAGCCAGTGTGGGAAGGTC	ACGTGCCAGGTCATCTTCAG
GAPDH	GGAGCGAGATCCCTCCAAAAT	GGCTGTTGTCATACTTCTCATGG

### 2.13 Statistical analysis

Statistical analysis was performed using the Statistical Analysis R software (version 4.4.3) and GraphPad Prism 9.0 (GraphPad Software, USA). The Wilcoxon rank-sum test was used to assess the differences between the groups. Pearson’s correlation analysis was used to evaluate the correlation between the hub genes and immune cell infiltration. Statistical significance was set at P< 0.05.

## 3 Results

### 3.1 Resource identification initiative

**Figure 2:**
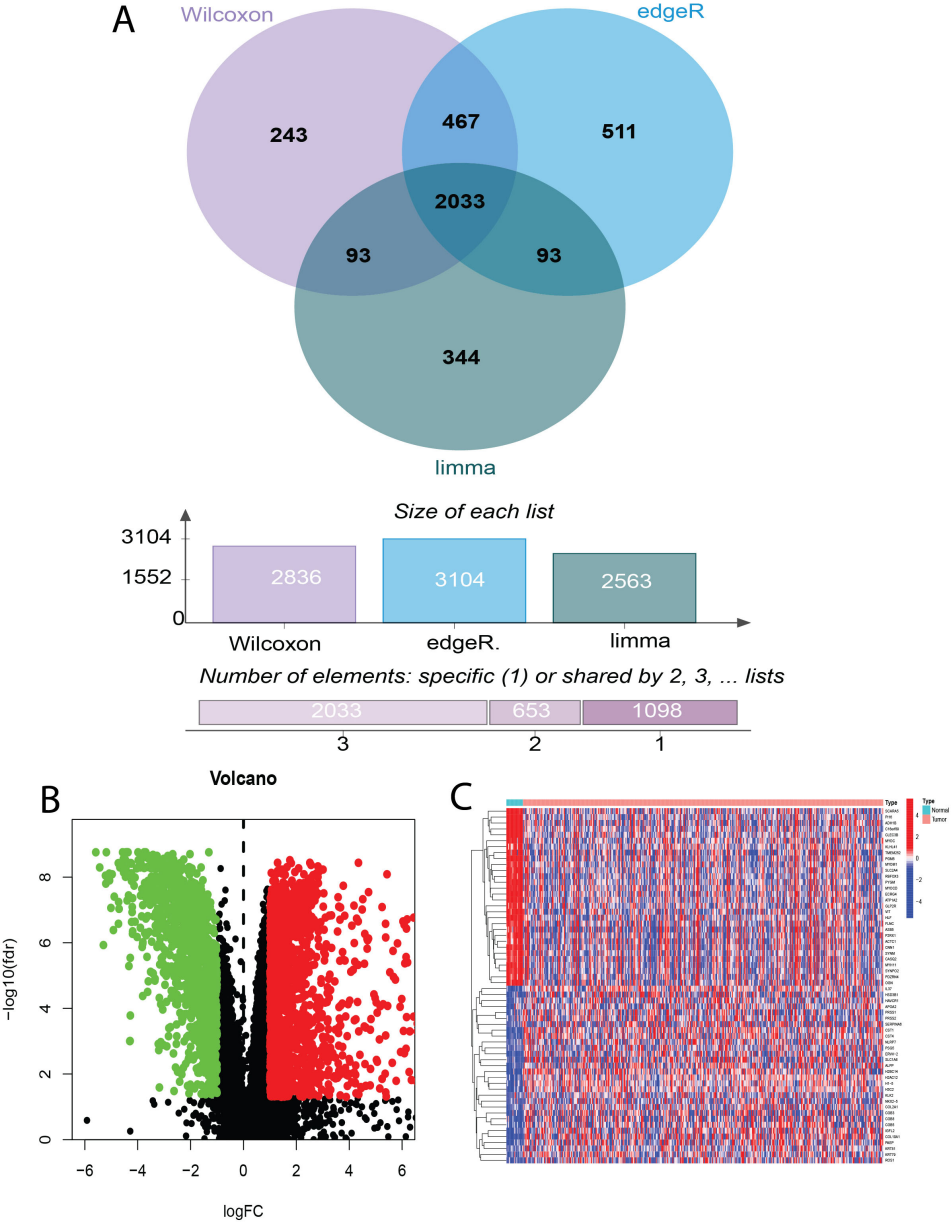
Identification of Differentially Expressed Genes (DEGs) in Bladder Cancer using Wilcoxon, edgeR, and limma Tests. **(A)** Venn diagram illustrating the overlap of DEGs identified by Wilcoxon rank-sum test, edgeR, and limma analysis in bladder cancer. **(B)** Volcano plot depicting bladder cancer DEGs and sample hierarchical clustering. **(C)** Heatmap of the top 30 up- and down-regulated DEGs.

To identify bladder cancer-associated genes, we performed differential expression analysis using three methods: edgeR, limma, and Wilcoxon test, comparing bladder cancer tissues with adjacent noncancerous control tissues. EdgeR identified 3104 differentially expressed genes (DEGs), comprising 1559 upregulated and 1545 downregulated genes. Limma identified 2563 DEGs (1084 upregulated and 1479 downregulated genes), whereas Wilcoxon test identified 2836 DEGs (1560 upregulated and 1276 downregulated genes). To enhance the reliability of DEG identification, we selected only genes identified by all three methods, resulting in a set of 2033 genes (**Figure 2A**). Of these, 881 genes were upregulated and 1152 were downregulated (**Figure 2B**). The top 30 upregulated and downregulated genes are shown in **Table 3** and **Figure 2C**, respectively. All identified DEGs exhibited a statistically significant adjusted P-value (adj.P-val) < 0.01, indicating statistically significant differences in expression. These DEGs provided a foundation for further functional and mechanistic studies.

**Table 3:** Top 30 upregulated and downregulated differentially expressed genes.

Top 30 Upregulated and Downregulated	
Upregulated	Downregulated
SCARA5, PI16, ADH1B, C16orf89, CLEC3B, MYOC, KLHL41, TMEM252, PGM5, MYOM1, SLC2A4, RBFOX3, PYGM, MYOCD, ECRG4, ATP1A2, GLP2R, VIT, HLF, FLNC, ASB5, P2RX1, ACTC1, CNN1, SYNM, CASQ2, MYH11, SYNPO2, PDZRN4, OGN	IL37, HSD3B1, HAVCR1, APOA2, PRSS1, PRSS2, SERPINA6, CST1, CST4, NLRP7, PSG5, ERVV−2, SLC1A6, ALPP, H2BC14, H2AC12, H1−5, H3C2, KLK2, NKX2−5, COL2A1, CGB3, CGB8, CGB5, IGFL2, COL10A1, PAEP, KRT81, KRT79, ROS1

### 3.2 Identification of bladder cancer immune-related genes using WGCNA

**Figure 3:**
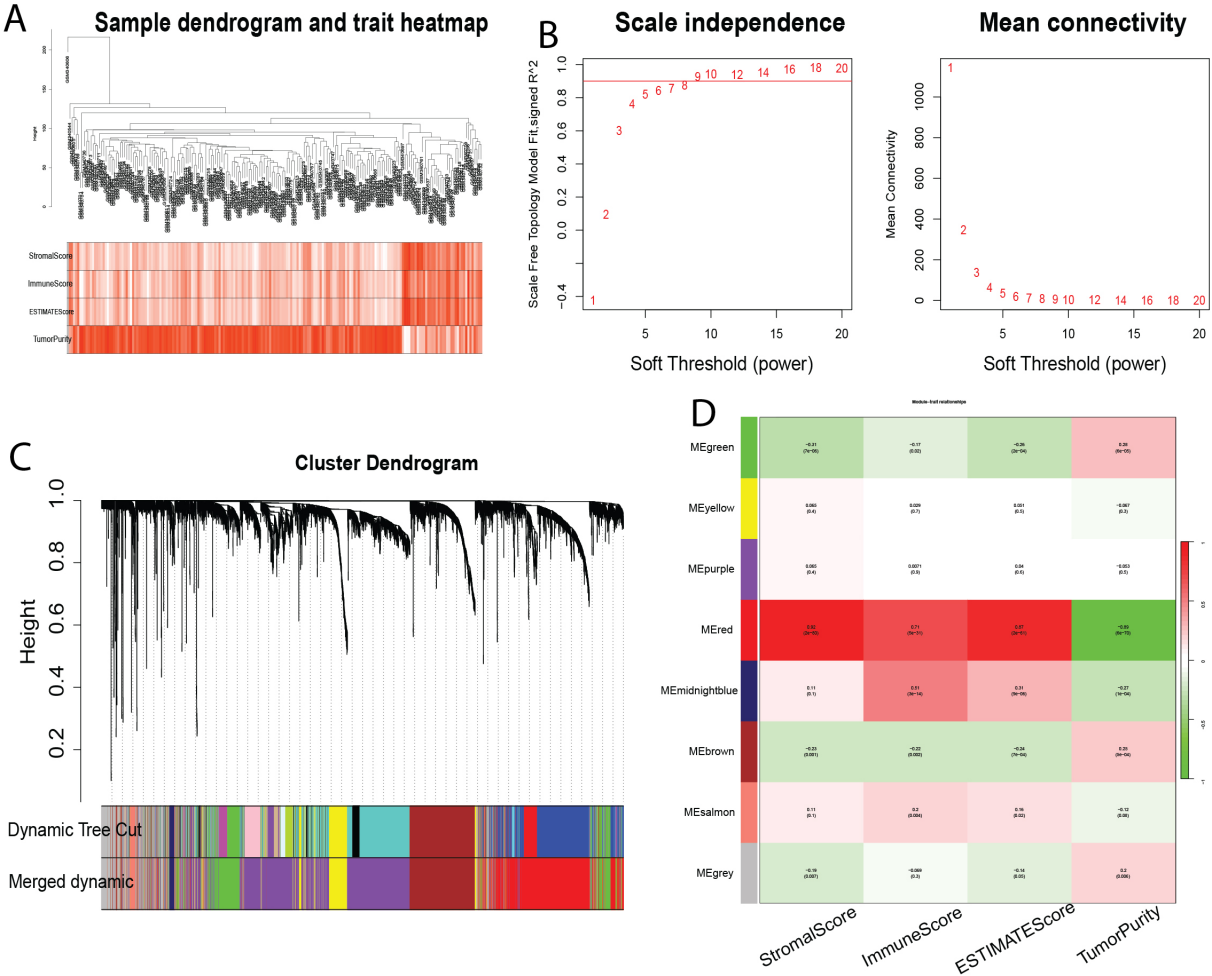
Identification of Modules Associated with Infiltrating Immune Cells using Weighted Gene Co-expression Network Analysis (WGCNA). **(A)** Sample clustering dendrogram. **(B)** Analysis of scale-free topology and mean connectivity for various soft-thresholding powers. **(C)** Dendrogram of all differentially expressed genes based on 1-Topological Overlap Matrix (TOM) dissimilarity measure. The color bands represent the results from dynamic tree cut analysis. **(D)** Heatmap showing the correlation between module eigengenes and infiltrating immune cell characteristics. The MEpurple module was selected for further analysis. TOM, topological overlap matrix; ME, module eigengene.

To identify genes associated with infiltrating immune cells within the bladder tumor microenvironment, we performed WGCNA analysis on the GSE13507 dataset. First, sample clustering analysis demonstrated that all 197 samples in the GSE21374 dataset were suitable for constructing a weighted gene co-expression network (**Figure 3A**). Subsequently, we set β = 8 (R² = 0.9) and merged modules with similarity < 0.6, resulting in a weighted gene co-expression network comprising eight modules (**Figures 3B, C**). Further analysis revealed correlations between these eight modules and StromalScore, ImmuneScore, ESTIMATE score, and Tumor Purity score (**Figure 3D**). The red module (MEred) exhibited the strongest positive correlation with the immune-related metrics. Furthermore, we calculated the correlation between genes in the MEred module and the four scores. Applying a threshold of correlation coefficient > 0.8 and P< 0.05, we identified 1391 genes associated with immune cells in the tumor immune microenvironment.

### 3.3 Causal Effects of genetically predicted eQTLs on bladder cancer and identification of candidate genes

**Figure 4:**
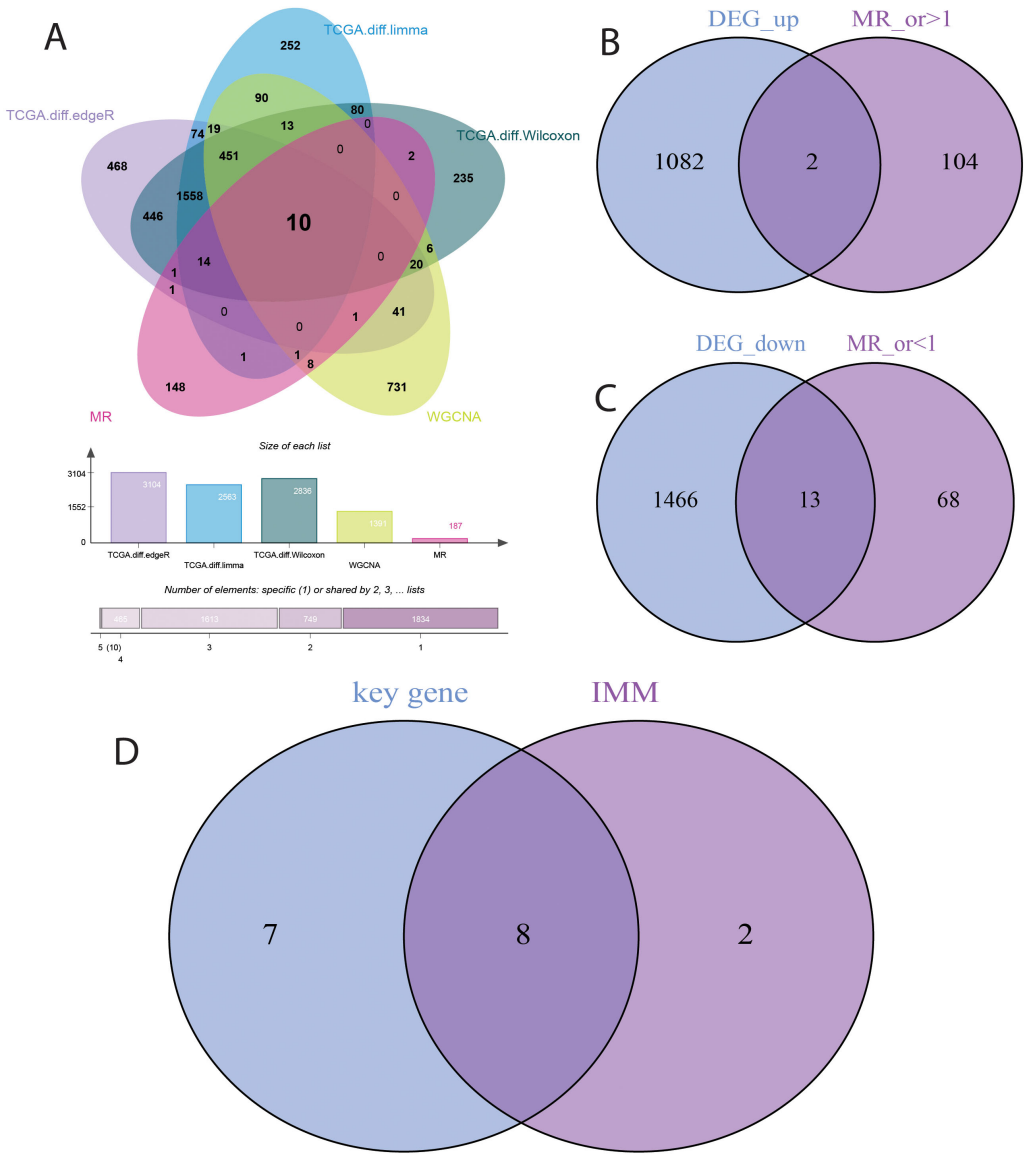
Selection of Key Genes in Bladder Cancer. **(A)** Venn diagram showing the overlap of 187 causally associated genes, 2033 differentially expressed genes (DEGs), and 1391 immune-related genes in bladder cancer. **(B)** Venn diagram showing the overlap between bladder cancer upregulated genes and genes with odds ratios (OR) > 1 from Mendelian randomization analysis. **(C)** Venn diagram showing the overlap between bladder cancer downregulated genes and genes with odds ratios (OR) < 1 from Mendelian randomization analysis. **(D)** Venn diagram showing the overlap between genes identified by Mendelian randomization causal analysis and immune-related genes in bladder cancer.

This study investigated the causal relationship between 5,430 expression quantitative trait loci (eQTLs) and bladder cancer risk. Based on the selection criteria of MR analysis, 187 eQTLs were causally associated with the development of bladder cancer. Integrating these 187 eQTLs with 2033 differentially expressed genes (DEGs) and 1391 immune-related genes, we identified 10 key genes (**Figure 4A**). Applying a filtering strategy—intersecting bladder cancer upregulated genes with genes exhibiting odds ratios (OR) > 1 in Mendelian randomization analysis, and bladder cancer downregulated genes with genes exhibiting OR < 1–we obtained two co-expressed upregulated genes and 13 co-expressed downregulated genes (**Figure 4B**). Finally, intersecting these key genes with risk-consistent genes yielded eight immune-related genes that were negatively associated with bladder cancer risk: LIMS2, TP53INP2, IRAK3, STX2, CYP27A1, IL11RA, KCNMB1, and PDLIM7 (**Figure 4C**).

### 3.4 Mendelian randomization analysis

**Figure 5:**
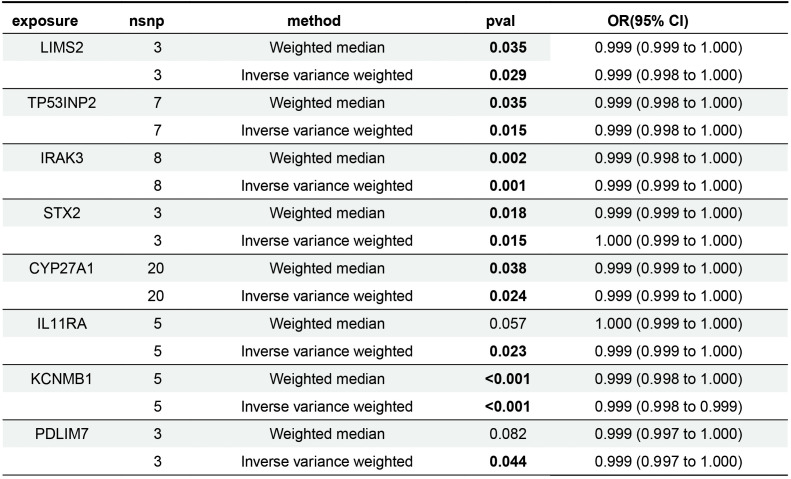
Forest plot of Mendelian randomization results for key gene.

Mendelian randomization analysis revealed that eight genes were negatively associated with bladder cancer risk (**Figure 5**). According to the IVW analysis, these genes and their associations were as follows: LIMS2 (OR = 0.9992; 95% CI, 0.9985-0.9999; P = 0.029); TP53INP2 (OR = 0.9989; 95% CI, 0.9980-0.9998; P = 0.015); IRAK3 (OR = 0.9991; 95% CI, 0.9986-0.9997; P = 0.001); STX2 (OR = 0.9995; 95% CI, 0.9991-0.9999; P = 0.015); CYP27A1 (OR = 0.9994; 95% CI, 0.9990-0.9999; P = 0.024); IL11RA (OR = 0.9995; 95% CI, 0.9990-0.9999; P = 0.023); KCNMB1 (OR = 0.9989; 95% CI, 0.9984-0.9995; P = 0.001); and PDLIM7 (OR = 0.9986; 95% CI, 0.9972-1.0000; P = 0.044). The directionality of these associations was further supported by the results of the Weighted Median (WM), MR-Egger, Simple Mode, and Weighted Mode analyses. Notably, the odds ratios (ORs) of these genes were very close to 1. In Mendelian randomization analysis, OR values close to 1 are expected, as continuous variables, such as gene expression (e.g., per-allele increase), typically have small effects on complex traits. In genetic studies of complex diseases, the effect size of a single-gene variant on disease risk is often minimal, making an OR close to 1 biologically plausible.

### 3.5 Sensitivity analyses

**Figure 6:**
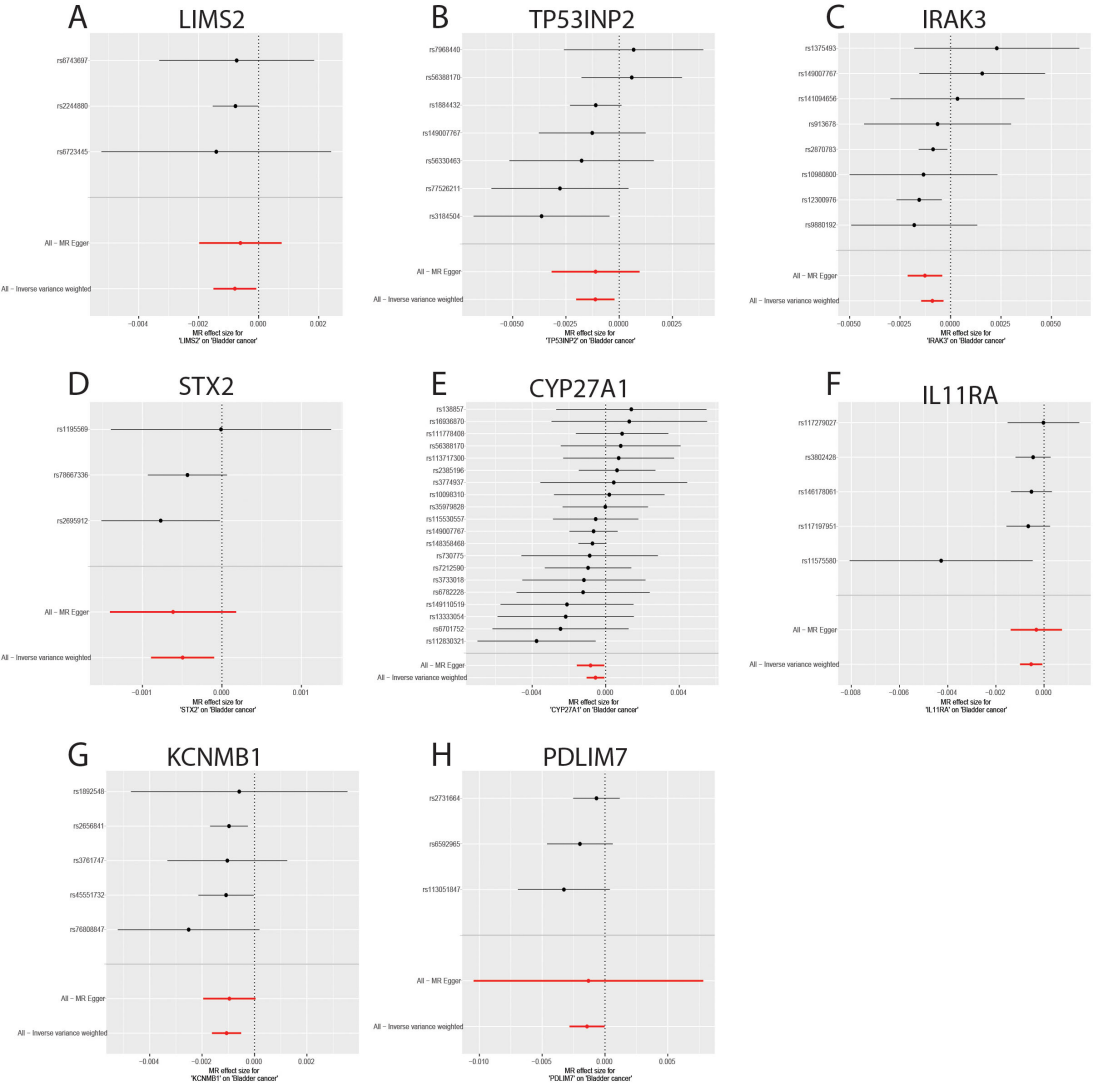
Forest plot of genes causally associated with bladder cancer. **(A-H)** MR effect size of key genes on bladder cancer; **(A)** LIMS2; **(B)** TP53INP2; **(C)** IRAK3; **(D)** STX2; **(E)** CYP27A1; **(F)** IL11RA; **(G)**; **(H)** PDLIM7.

The sensitivity analyses revealed no evidence of significant heterogeneity or horizontal pleiotropy (**Table 4**). Cochran’s Q test was used to assess the heterogeneity among instrumental variable estimates derived from individual genetic variants. The use of a random-effects inverse variance-weighted (IVW) model mitigated the influence of heterogeneity on the results. The dispersion of causal associations presented in the funnel plot suggests the absence of confounding bias in the causal effects of eQTL expression levels on bladder cancer (**Supplementary Figure S1**). Furthermore, an MR-Egger intercept test with a P-value > 0.05 confirmed the absence of horizontal pleiotropy (**Supplementary Figure S2**). The leave-one-out sensitivity analysis demonstrated that excluding a single SNP did not significantly alter the MR analysis results, thereby ensuring the robustness of the findings (**Supplementary Figure S3**). A forest plot showed the risk associations between these eight genes and bladder cancer (**Figure 6**).

**Table 4:** Tests for heterogeneity and pleiotropy between crossover genes and bladder cancer.

exposure	Heterogeneity testing						pleiotropy		
	Inverse variance-weighted			MR-Egger			MR-Egger		
	Q	Q_df	Q_P	Q	Q_df	Q_P	intercept/x-4	SE/x-4	P
LIMS2	0.105	2	0.949	0.010	1	0.921	-0.559	1.807	0.809
TP53INP2	6.771	6	0.343	6.771	5	0.238	-0.016	1.492	0.992
IRAK3	7.009	7	0.428	5.780	6	0.448	1.000	0.906	0.310
STX2	1.052	2	0.591	0.934	1	0.334	0.634	1.849	0.790
CYP27A1	13.022	19	0.837	12.158	18	0.839	0.5.95	0.640	0.365
IL11RA	4.290	4	0.368	4.014	3	0.260	-0.179	3.934	0.681
KCNMB1	1.218	4	0.875	1.152	3	0.765	-0.393	1.520	0.813
PDLIM7	1.786	2	0.409	1.785	1	0.182	-0.156	6.108	0.984

Q, heterogeneity statistic; Q_df, degrees of freedom; MR, Mendelian randomization; SE, standard deviation; Q_P>0.05, no heterogeneity; P>0.05, no level of multivariate validity.

### 3.6 GO and KEGG enrichment analysis of shared genes

The chromosomal locations of eight key genes were mapped and visualized (**Supplementary Figure S4A**). Subsequently, GO and KEGG enrichment analyses were performed on these eight genes to elucidate their associated biological processes and pathways. In terms of biological processes, these genes are primarily involved in vitamin D biosynthesis and the Toll signaling pathways. Cellular component analysis localized the genes in the focal adhesion, leading edge, and cell-substrate junction. Molecular function analysis primarily highlighted calcium-activated potassium channel activity and muscle alpha-actinin binding (**Supplementary Figures S4B–D**). KEGG pathway analysis showed a significant involvement in primary bile acid biosynthesis (**Supplementary Figures S4E, F**).

### 3.7 Immune infiltration analysis

**Figure 7:**
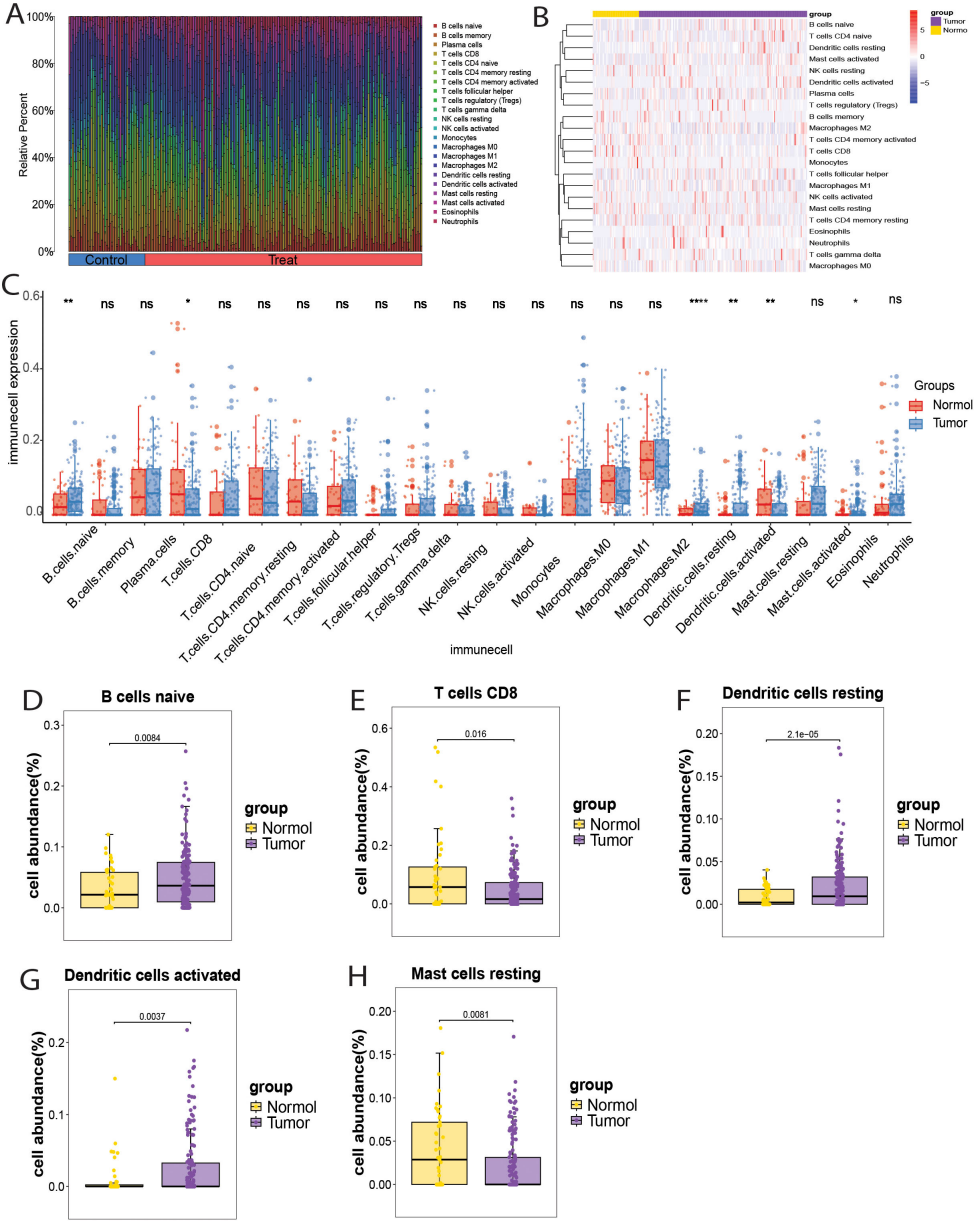
Immune cell infiltration landscape in bladder cancer. **(A)** Bar chart showing the distribution of 22 immune cell types in each sample. **(B)** Heatmap showing the expression of 22 immune cell types in bladder cancer and normal samples. **(C)** Box plot showing the expression of 22 immune cell types in bladder cancer and normal samples. **(D-H)** Box plots showing the differential infiltration landscape of six immune cell types in bladder cancer and normal samples: **(D)** Naive B cells; **(E)** CD8+ T cells; **(F)** Resting dendritic cells; **(G)** Activated dendritic cells; **(H)** Resting mast cells. Each p-value is shown above the corresponding box plot (NS: p > 0.05; *: p ≤ 0.05; **: p ≤ 0.01;****:p ≤ 0.0001).

The merged GEO datasets (GSE31189, GSE31684, and GSE37815) were used for CIBERSORT analysis of bladder cancer, and the results were further supported by xCell analysis. First, samples with a CIBERSORT P-value < 0.05 were excluded, resulting in 163 tumor tissue samples and 45 normal tissue samples. The composition and proportion of infiltrating immune cells in each sample were visualized using bar plots and heat maps (**Figures 7A, B**). Analysis of the infiltration landscape of 22 immune cell types revealed that six immune cells showed significantly different expression between bladder cancer and normal tissues. Among them, eosinophils showed low infiltration abundance in both the tumor and normal groups (P < 0.05) and were excluded from further analysis (**Figure 7C**). Naive B cells, resting dendritic cells, and activated dendritic cells were upregulated in tumor tissues, whereas CD8+ T cells and resting mast cells were downregulated (**Figures 7D–H**). The immune infiltration landscape of 64 immune cell types in bladder cancer and normal tissues analyzed by the xCell method is shown in **Supplementary Figures S5A, B**. Although the xCell results did not show statistically significant differences (P < 0.05), they demonstrated the same trends as the CIBERSORT analysis: upregulation of naive B cells, resting dendritic cells, and activated dendritic cells and downregulation of CD8+ T cells and resting mast cells in tumor tissues (**Supplementary Figure S5C**). Therefore, naïve B cells, resting dendritic cells, activated dendritic cells, CD8+ T cells, and resting mast cells may play key roles in bladder cancer pathogenesis.

### 3.8 Correlation between immune-related genes and immune cells

**Figure 8:**
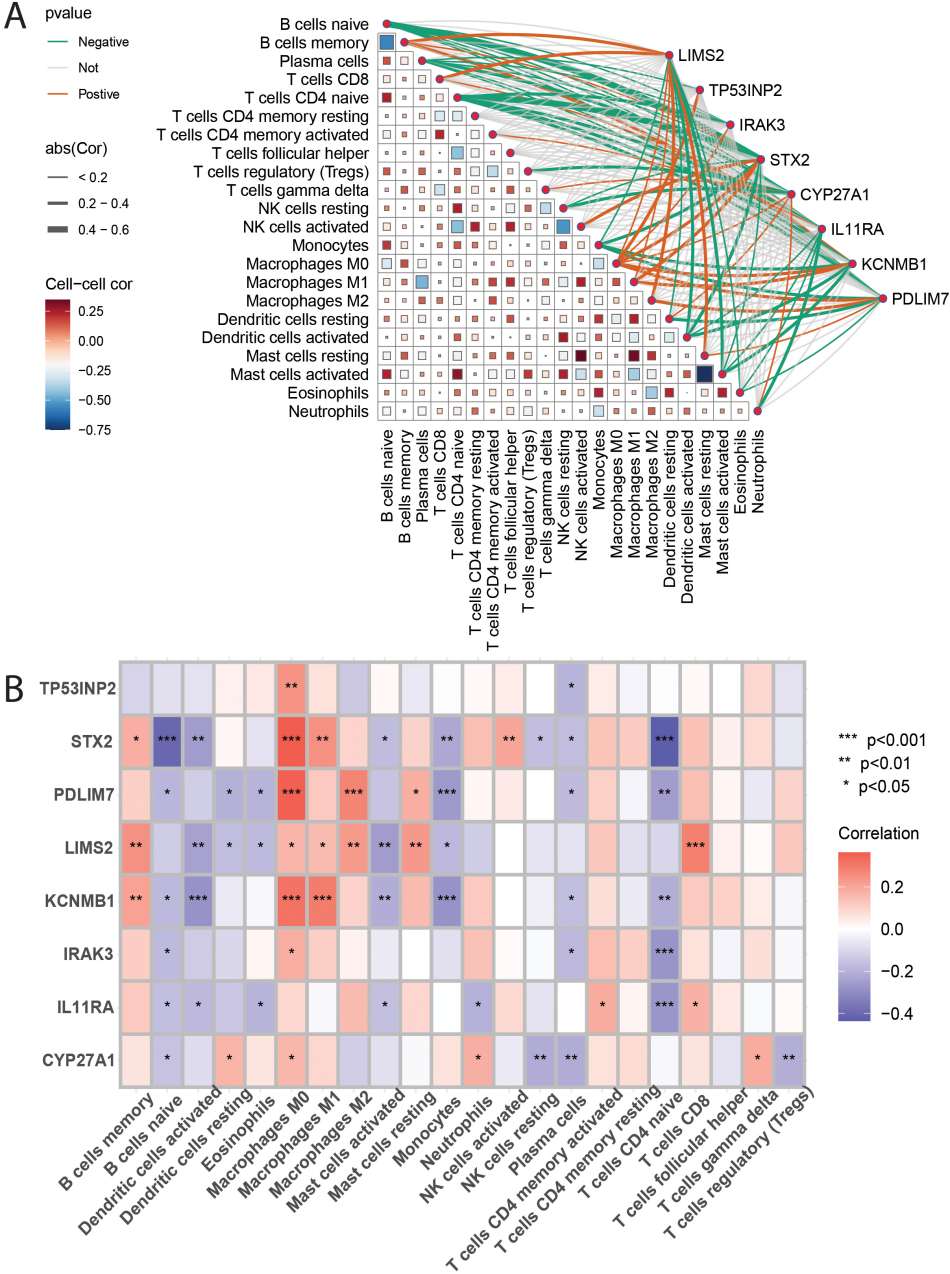
**(A, B)**: These figures present heatmaps illustrating the correlation analysis between 22 immune cell types and the eight key genes. **Figure 9A** likely shows correlations between the immune cells themselves, and **Figure 9B** likely shows the correlations between the immune cells and the eight key genes, providing a visual representation of the described relationships.

To investigate the association between eight key genes (TP53INP2, STX2, PDLIM7, LIMS2, KCNMB1, IRAK3, IL11RA, CYP27A1) and immune cells, Spearman rank correlation analysis was used to examine their correlation with different immune cell subsets (**Figures 8A, B**). The results showed that: TP53INP2 gene expression was positively correlated with M0 macrophages and negatively correlated with plasma cells; STX2 gene expression was positively correlated with memory B cells, M0 and M1 macrophages, and activated NK cells, and negatively correlated with naive B cells, activated dendritic cells, activated mast cells, monocytes, resting NK cells, plasma cells, and naive CD4+ T cells; PDLIM7 gene expression was positively correlated with M1 and M2 macrophages and resting mast cells, and negatively correlated with naive B cells, naive dendritic cells, eosinophils, monocytes, plasma cells, and naive CD4+ T cells; LIMS2 gene expression was positively correlated with B cells memory, M0/M1/M2 macrophages, resting mast cells, and CD8+ T cells, and negatively correlated with activated and resting dendritic cells, eosinophils, and monocytes; KCNMB1 gene expression was positively correlated with memory B cells, M0 and M1 macrophages, and negatively correlated with naive B cells, activated dendritic cells, activated mast cells, monocytes, plasma cells, and naive CD4+ T cells; IRAK3 gene expression was positively correlated with M0 macrophages and negatively correlated with naive B cells, plasma cells, and naive CD4+ T cells; IL11RA gene expression was positively correlated with activated CD4+ memory T cells and CD8+ T cells, and negatively correlated with naive B cells, activated dendritic cells, eosinophils, activated mast cells, neutrophils, and naive CD4+ T cells; CYP27A1 gene expression was positively correlated with resting dendritic cells, M0 macrophages, neutrophils, and γδ T cells, and negatively correlated with naive B cells, resting NK cells, plasma cells, and regulatory T cells (Tregs). The results showed that STX2, PDLIM7, LIMS2, and KCNMB1 were positively correlated with the infiltration levels of memory B cells, macrophages M0/M1/M2, resting mast cells, and CD8+ T cells, with M0 macrophages exhibiting the strongest correlation with these four genes. In contrast, the infiltration levels of naive B cells, activated dendritic cells, monocytes, plasma cells, and naive CD4+ T cells were significantly negatively correlated with STX2, PDLIM7, LIMS2, KCNMB1, and IRAK3. Naive CD4+ T cells showed the most pronounced negative correlations with all five genes. These findings suggest that STX2, PDLIM7, LIMS2, KCNMB1, and IRAK3 may influence tumor progression and patient prognosis by modulating immunosuppressive infiltration and immune evasion within the tumor microenvironment.

### 3.9 Evaluation and validation of the potential value of key genes

**Figure 9:**
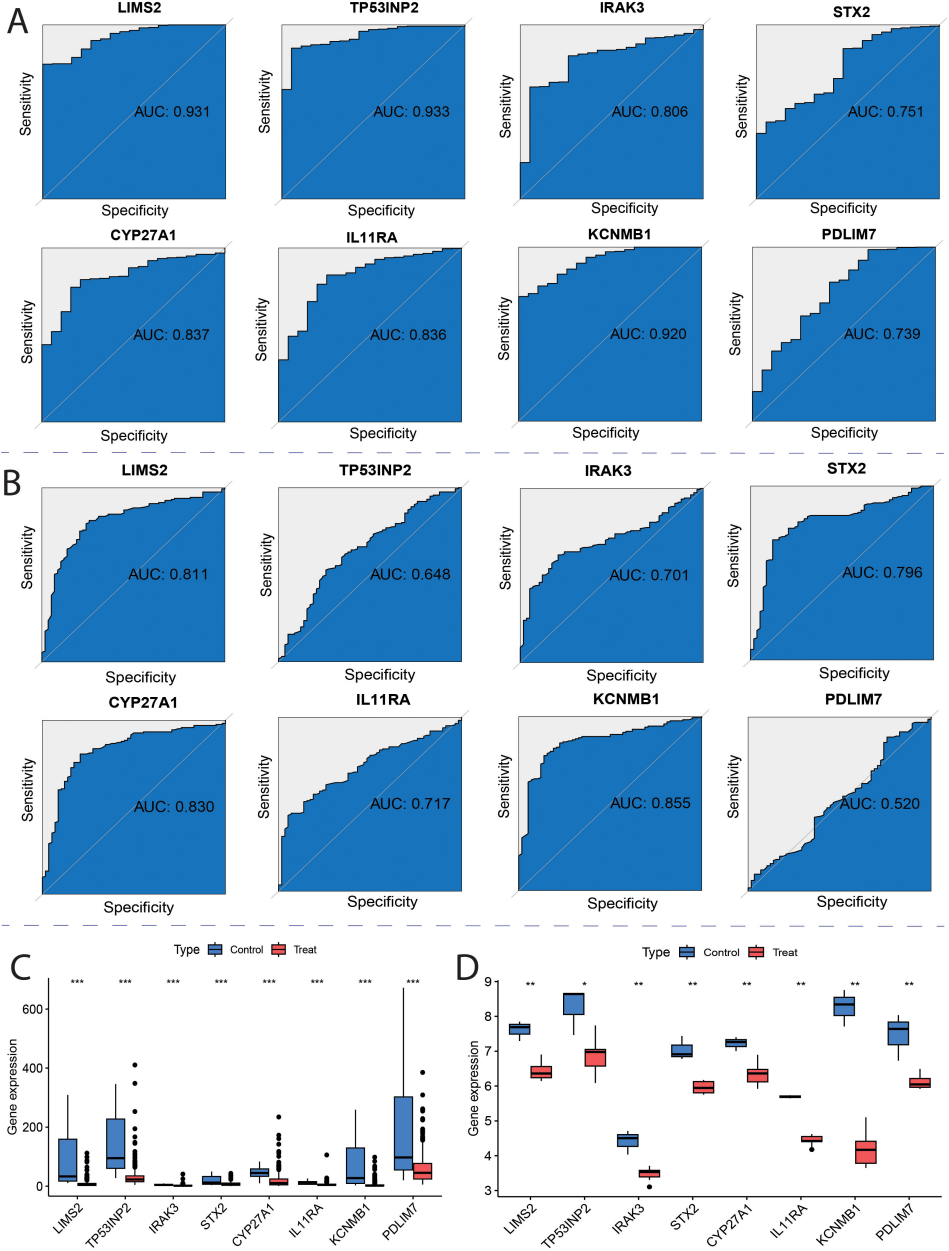
ROC curves for key genes. **(A)** Receiver Operating Characteristic (ROC) curves of hub genes in the TCGA; **(B)** ROC curves of hub genes in the GSE13507; **(C)** TCGA Characterized Gene Intersection Single Gene Expression Boxplot; **(D)** GSE7476 Characterized Gene Intersection Single Gene Expression Boxplot. Each p-value is shown above the corresponding box plot (NS: p > 0.05; *: p ≤ 0.05; **: p ≤ 0.01; ***: p ≤ 0.001).

To evaluate the diagnostic potential of the eight key genes in distinguishing tumor from non-tumor samples, receiver operating characteristic (ROC) curve analysis was performed using TCGA and GSE13507 bladder cancer datasets. TCGA analysis showed that all eight genes had an area under the curve (AUC) greater than 0.7 (**Figure 9A**), indicating good diagnostic potential for distinguishing between normal and tumor tissues. Furthermore, analysis of the GSE13507 dataset revealed that six genes, LIMS2, IRAK3, STX2, CYP27A1, IL11RA, and KCNMB1, had AUC values greater than 0.7 (**Figure 9B**), demonstrating good diagnostic potential in differentiating healthy adjacent tissues from tumor samples. These results indicate that all eight genes exhibit strong discriminatory ability between bladder cancer and normal tissues, with LIMS2, IRAK3, STX2, CYP27A1, IL11RA, and KCNMB1 showing greater potential diagnostic value. To validate the expression levels of the eight key genes (LIMS2, TP53INP2, IRAK3, STX2, CYP27A1, IL11RA, KCNMB1, and PDLIM7) in tumor and non-tumor samples, we analyzed their expression in TCGA and GSE7476 datasets. We observed that in both TCGA (training) and GSE7476 (validation) datasets, these eight key genes were significantly downregulated in tumor samples, consistent with Mendelian randomization results (**Figures 9C, D**). Therefore, LIMS2, TP53INP2, IRAK3, STX2, CYP27A1, IL11RA, KCNMB1, and PDLIM7 have been proposed as potential biomarkers of bladder cancer.

### 3.10 Validation of key genes by real-time quantitative PCR

**Figure 10:**
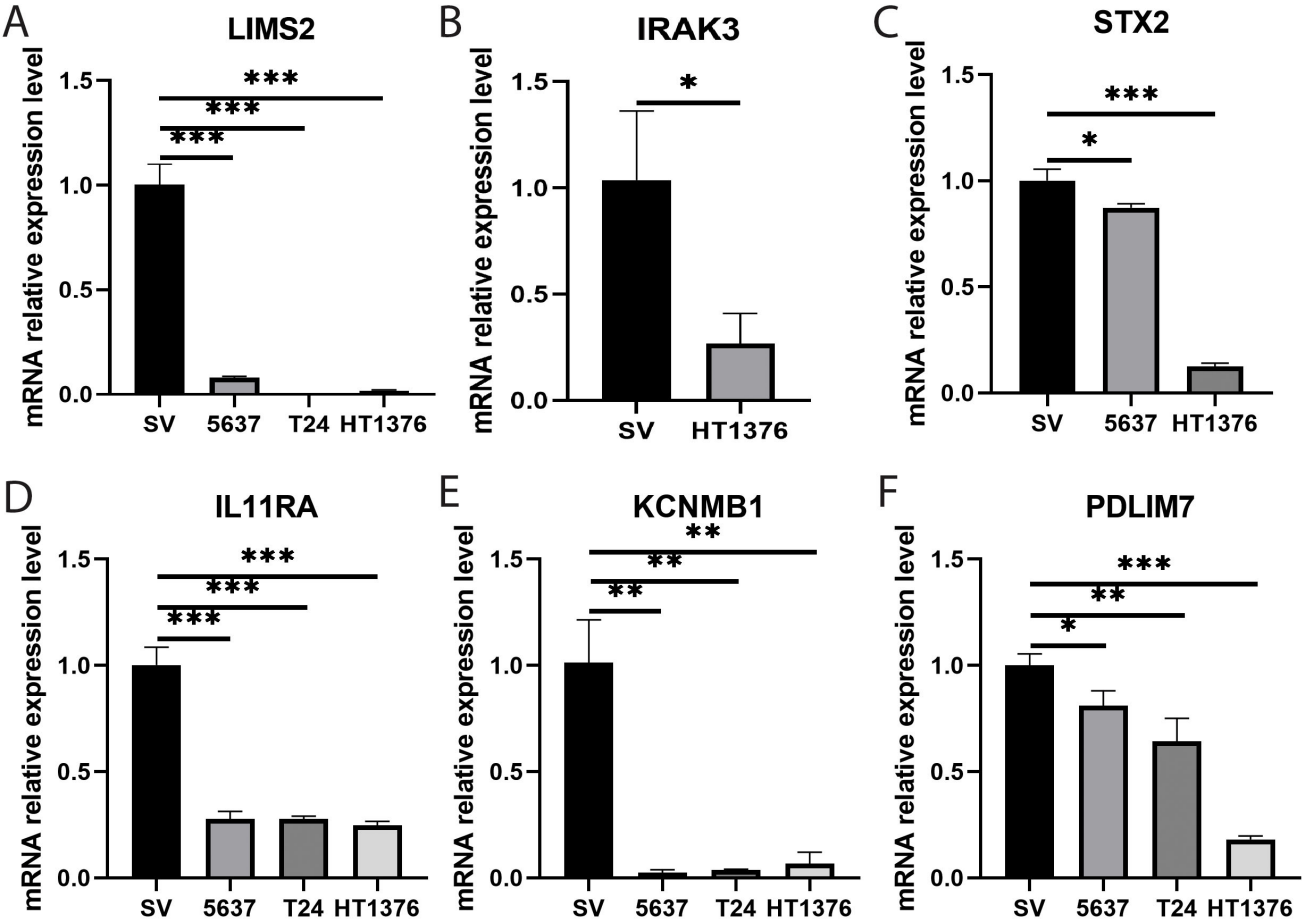
Bar graphs showing qRT-PCR expression and differential analysis of key genes. **(A-F)** Bar graphs showing differential expression analysis of eight key genes **(A)** LIMS2; **(B)** IRAK3; **(C)** STX2; **(D)** IL11RA; **(E)** KCNMB1; and **(F)** PDLIM7 in bladder normal cell line SV and bladder cancer cell lines 5637, T24, and HT1376. Differences between groups were assessed using the Wilcoxon rank-sum test. Each p-value is shown above the corresponding bar (NS: P > 0.05; *: P ≤ 0.05; **: P ≤ 0.01; ***: P ≤ 0.001).

To further investigate the potential roles of the eight key genes (LIMS2, TP53INP2, IRAK3, STX2, CYP27A1, IL11RA, KCNMB1, and PDLIM7) in bladder tumorigenesis, we performed qRT-PCR to quantify their expression levels in the bladder normal cell line SV and bladder cancer cell lines 5637, T24, and HT1376. Differential expression analysis revealed that, compared to the normal bladder cell line SV, the expression levels of LIMS2, IL11RA, KCNMB1, and PDLIM7 were significantly downregulated in all three bladder cancer cell lines (5637, T24, and HT1376). Furthermore, IRAK3 expression was significantly downregulated in HT1376 cells, whereas STX2 was significantly downregulated in 5637 and HT1376 cells (**Figure 10**). Subsequent box plot analysis further demonstrated significantly lower expression of six genes (LIMS2, IRAK3, STX2, IL11RA, KCNMB1, and PDLIM7) in bladder cancer cell lines (**Supplementary Figure S7**), consistent with bioinformatics analysis. This strongly supports the conclusion that these genes play crucial regulatory roles in bladder cancer development. These findings provide important experimental evidence for further investigation of the specific mechanisms of these genes in the immune microenvironment of bladder cancer and their potential as diagnostic and therapeutic targets for bladder cancer.

## 4 Discussion

Seven independent datasets were integrated from TCGA, GEO, and GWAS databases, and a multi-source transcriptomic combined Mendelian randomization analysis strategy was employed to identify eight immune-related key genes: LIMS2, TP53INP2, IRAK3, STX2, CYP27A1, IL11RA, KCNMB1, and PDLIM7. This study confirmed that these eight genes were significantly downregulated in bladder cancer tissues and their expression levels were significantly negatively correlated with bladder cancer risk, suggesting that these genes may participate in the development of bladder cancer by regulating immune pathways. Immune infiltration analysis using the CIBERSORT algorithm revealed that the proportions of naive B cells, resting dendritic cells, and activated dendritic cells were significantly increased in tumor tissues, whereas the infiltration levels of CD8+ T cells and resting mast cells were significantly decreased. Correlation analysis showed that M0 macrophages had the strongest positive correlation with the expression of STX2, PDLIM7, LIMS2, and KCNMB1 (P < 0.05), whereas the infiltration levels of naive CD4+ T cells were significantly negatively correlated with the expression of STX2, PDLIM7, LIMS2, KCNMB1, and IRAK3 (P < 0.05). Diagnostic performance evaluation demonstrated that all eight genes exhibited a high diagnostic discrimination ability (AUC > 0.7). The consistent downregulation of these genes in bladder cancer tissues was further validated using independent public datasets, and qRT-PCR experiments confirmed the differential expression of the following six genes: LIMS2, IRAK3, STX2, IL11RA, KCNMB1, and PDLIM7. Importantly, these findings provide candidate biomarkers for early diagnosis and treatment of bladder cancer, which may help improve patient prognosis and guide personalized therapeutic strategies.

Gene ontology (GO) and Kyoto Encyclopedia of Genes and Genomes (KEGG) enrichment analyses revealed that the eight key genes were significantly enriched in signaling pathways such as focal adhesion. Notably, focal adhesion kinase (FAK) plays a critical role in regulating tumor growth, immune suppression, metastasis, and therapeutic resistance. Its mechanism of promoting tumor progression by modulating crosstalk within the tumor immune microenvironment (TME) makes it a potential target for anticancer therapy (35). The involvement of bile acid metabolic pathways in tumor immune regulation (36) aligns with our KEGG results, suggesting that bile acid signaling may represent a novel direction for tumor immunotherapy. Additionally, the gene set was enriched in cellular structure-related pathways, such as the cell leading edge and cell–substrate junction, further supporting the regulatory roles of the identified genes in tumor-immune interactions. By integrating the CIBERSORT and xCell algorithms, this study systematically characterized the immune cell infiltration landscape in bladder cancer tissues. The proportions of naive B cells and resting/activated dendritic cells (DCs) were significantly increased in tumor tissues, whereas infiltration of CD8+ T cells and resting mast cells was decreased. It is noteworthy that although some comparisons in the xCell analysis did not reach statistical significance (P > 0.05), their trends were consistent with the CIBERSORT results, providing important clues for the regulatory mechanisms of the immune microenvironment (33, 37). Of particular importance, this study found significant enrichment of naive B cells in the tumor microenvironment. As a B cell subset that has not undergone antigen stimulation, these cells can differentiate into various functional subtypes such as regulatory B cells (Bregs) (37), which play important roles in the TME. These subtypes exhibit diverse biological functions and influence tumor prognosis (38). The observed enrichment of naive B cells suggests that they may promote bladder cancer progression by inducing immunosuppressive Breg differentiation, a mechanism that warrants further functional validation.

The characteristics of immune cell infiltration observed in this study have significant pathological implications. As the most effective antigen-presenting cells (39), dendritic cells (DCs) exhibit notable changes in bladder tumors, with both resting DCs (immature state) and activated DCs (mature state) showing significantly increased proportions. This observation is consistent with the results of previous studies (40). The underlying mechanism may involve tumor-associated DCs secreting immunosuppressive factors such as IL-10 and TGF-β1, which inhibit T cell activation and promote tumor growth (41), suggesting that DC dysfunction may be a key component of immune evasion. Meanwhile, the core effector T cell subset, CD8+ T cells, was significantly reduced in the tumor tissues. This finding has important pathological significance, as CD8+ T cells are the primary effector immune cells responsible for tumor immune surveillance (42), and their reduction directly weakens the antitumor immune response. Notably, this study also observed a decrease in resting mast cells, which can actively eliminate tumor cells and prevent tumorigenesis through cytokines, such as IL-1, IL-4, IL-6, and TNF-α. A reduction in resting mast cells may disrupt stromal homeostasis and antitumor balance (43). It is worth emphasizing that although the upregulation of activated DCs and naive B cells may reflect a compensatory enhancement of antigen presentation, the exhaustion of CD8+ T cells and loss of mast cells together shape an immunosuppressive microenvironment. This immunosuppressive milieu is closely associated with resistance to PD-1 inhibitors and poor prognosis in bladder cancer patients (44).

This study systematically revealed the potential regulatory roles of naive B cells, resting dendritic cells, activated dendritic cells, CD8+ T cells, and resting mast cells in the immune microenvironment of bladder cancer. To overcome the current technical limitations, future research should integrate genome-wide association studies (GWAS) with single-cell RNA sequencing (scRNA-seq) of immune cells to precisely identify the specific immune cell types that mediate the genetic risk of bladder cancer. This approach has shown significant advantages in the study of complex diseases, and previous studies have successfully identified the key immune cell types associated with COVID-19 by integrating GWAS and scRNA-seq data (45, 46). Similarly, Li et al. applied this strategy to identify genetic regulatory cell types associated with the gut microbiome (47). Although this study delineated the immune infiltration landscape in bladder cancer, subsequent research should combine GWAS, single-cell sequencing technologies, and experimental validation to elucidate the potential for deeper investigation of the causal links between immune-related genes, pathways, and immune cell types in bladder cancer.

LIM zinc finger domain-containing 2 (LIMS2) is a focal adhesion protein containing five LIM domains (48) that interacts with integrins and various other proteins, and plays a crucial role in cell signaling pathways (49). Its homolog, LIMS1, forms a LIMS1-ILK-Parvin complex with integrin-linked kinase (ILK) and Parvin proteins and participates in the critical regulation of various cancers. Notably, studies have shown that LIMS2 can bind to ILK, forming a LIMS2-ILK-Parvin complex, and competitively inhibits the binding of LIMS1 to ILK (48), thereby affecting its function in tumorigenesis. Previous studies have observed that LIMS2 silencing significantly enhances the migratory ability of gastric cancer cells (50), In this study, by integrating transcriptomic data with Mendelian randomization (MR) analysis, we found that LIMS2 expression was significantly downregulated in bladder cancer tissues, consistent with the expression pattern reported in gastric cancer. Notably, the MR results suggested a causal association between LIMS2 expression and bladder cancer risk. Combined with the immune infiltration analysis showing a significant positive correlation between LIMS2 and CD8+ T cell infiltration, this indicates that LIMS2 may regulate the immune microenvironment of bladder tumors. Although there are currently no clinical trials directly targeting LIMS2, its binding partner ILK has been identified as a potential therapeutic target in ovarian cancer (51). Notably, LIMS2 loss may lead to an imbalance in the ILK/Parvin complex, a pathological state that could potentially be functionally compensated by CRISPR-Cas9–mediated gene replacement therapy or synthetic LIM domain-mimicking peptides, providing a theoretical basis for developing novel targeted strategies.

The tumor protein p53 inducible nuclear protein 2 (TP53INP2) encodes a protein involved in regulating autophagy, which is crucial for maintaining normal autophagosome formation and maturation (52). TP53INP2 not only plays a key role in cellular metabolism but also regulates tumor cell invasiveness and migration (53). Furthermore, TP53INP2 has been identified as a potential biomarker for thyroid cancer (54). In bladder cancer, TP53INP2 influences cell migration, invasion, and epithelial-mesenchymal transition (EMT) by regulating the GSK-3β/β-catenin/Snail1 pathway (55). Previous studies have shown that TP53INP2 expression is significantly decreased in colorectal cancer tissues, and its silencing promotes tumor cell proliferation (56). Mendelian randomization (MR) analysis revealed a negative causal association between TP53INP2 expression levels and bladder cancer risk. Combined with immune infiltration analysis showing a positive correlation between TP53INP2 expression and M0 macrophage infiltration, these findings suggested that this gene may influence the tumor immune microenvironment by regulating tumor-associated macrophages (TAMs). Notably, autophagy can prevent tumor proliferation and metastasis in early stage cancers, and autophagy deficiency has been shown to disrupts T-cell homeostasis and anti-tumor immune responses (57). Given that TAMs are the most abundant immune cell population in the tumor microenvironment (58), and that autophagy regulates their polarization toward the M2 phenotype (59), this study proposes that downregulation of TP53INP2 may modulate bladder cancer development by influencing macrophage polarization. Although no clinical interventions targeting TP53INP2 have yet been established, its unique mechanism of regulating bladder cancer progression via the autophagy–macrophage axis makes it a potential target for immunotherapy.

Interleukin-1 receptor-associated kinase 3 (IRAK3) is a key regulatory molecule in the inflammatory response of the innate immune system that plays a crucial role in maintaining immune homeostasis (60). This study found that IRAK3 expression was downregulated in bladder cancer tissues, a pattern highly consistent with that observed in prostate cancer (61). Mechanistic studies have shown that promoter methylation of IRAK3 is a key epigenetic mechanism underlying its silencing, a phenomenon not only observed in hepatocellular carcinoma (62), but also reflected by increased methylation levels negatively correlated with gene expression in gliomas (63). Notably, Mendelian randomization (MR) analysis in this study confirmed a significant negative association between IRAK3 expression and bladder cancer risk, and its expression level was positively correlated with M0 macrophage infiltration in the tumor microenvironment, suggesting that IRAK3 may regulate the bladder tumor immune microenvironment. Accumulating evidence supports that IRAK3 expression in tumor-associated macrophages impairs cancer cell immune surveillance while effectively preventing excessive inflammation that drives cancer progression. Enhancing the host immune response in IRAK3-deficient mice can inhibit the growth of transplanted cancer cells (64), highlighting the critical role of IRAK3 in tumor immune regulation. Although no direct clinical interventions targeting IRAK3 currently exist, its potential value in bladder cancer should not be overlooked, and the gene remains a promising candidate for therapeutic development in bladder cancer treatment.

Syntaxin 2 (STX2), a highly conserved member of the syntaxin family (65), exerts its function through distinct domains: a C-terminal domain responsible for membrane anchoring, and an N-terminal domain mediating molecular interactions and signal transduction (66). The synaptosome-associated protein family plays an important role as membrane vesicle transport receptors in intracellular vesicle trafficking and secretion (67). Previous studies demonstrated that STX2 participates in the occurrence, development, and metastasis of various cancers by regulating the expression of key oncogenes such as β-catenin and MMP9 (68–70). The results of this study indicate that STX2 is significantly downregulated in bladder cancer tissues, and its expression level is negatively correlated with disease risk, suggesting that STX2 may function as a tumor suppressor gene. As a key regulator of membrane vesicle transport and secretion, STX2’s biological role is closely related to that of the exosomes. Previous studies have confirmed that exosomes play important regulatory roles in tumor initiation, invasion, and metastasis, and exosome-mediated immune modulation has garnered increasing attention (71). An integrated analysis revealed a significant positive correlation between STX2 expression and M0 macrophage infiltration. Combined with its role in exosome regulation, STX2 may influence the immune microenvironment of bladder cancer by modulating exosome secretion. Notably, prior research has identified STX2 as a potential therapeutic target and biomarker in colorectal cancer (72), Building on these findings, this study further elucidates its immunoregulatory role in bladder cancer. Although there are currently no direct clinical interventions targeting STX2, this gene still holds the potential for development as a bladder cancer–related biomarker.

Cytochrome P450 family 27 subfamily A member 1 (CYP27A1) belongs to the cytochrome P450 superfamily of enzymes and catalyzes 27-hydroxylation of cholesterol to produce 27-hydroxycholesterol (27-HC) (73). 27-HC can influence tumor cell proliferation, differentiation, and apoptosis by regulating cellular cholesterol homeostasis (74, 75). Studies have shown that CYP27A1 is downregulated in prostate cancer tissues, and restoration of its expression inhibits prostate cancer cell growth by increasing 27-HC production (76). The results of this study showed that CYP27A1 is significantly downregulated in bladder cancer tissues, and its expression level is negatively correlated with disease risk, which is consistent with the findings reported in prostate cancer. Previous studies confirmed that CYP27A1 catalyzes the production of 27-hydroxycholesterol (27-HC) from cholesterol and exhibits a significant growth inhibitory effect in renal cancer cells (77). Research indicates that CYP27A1 overexpression promotes 27-HC production and significantly suppresses bladder cancer cell growth (78), suggesting that CYP27A1 overexpression may represent a potential therapeutic strategy for bladder cancer. Combined with the immune infiltration analysis, CYP27A1 expression was significantly positively correlated with the infiltration level of resting dendritic cells (r = 0.42, P = 0.001), implying that this gene may influence disease progression by modulating the tumor immune microenvironment. Based on the findings of this study and the existing literature, we speculated that CYP27A1 might affect the development of bladder cancer by regulating 27-HC levels. Although no clinical therapies directly targeting CYP27A1 currently exist, its regulatory role in the tumor microenvironment suggests its potential value as a predictive biomarker for targeted therapy efficacy in bladder cancer.

Interleukin 11 receptor subunit alpha (IL11RA) encodes a cytokine receptor produced by stromal cells. As a member of the pleiotropic and redundant cytokine family, signal transduction depends on high-affinity binding to the gp130 signaling subunit (79). Extensive research indicates that IL-11 and its receptor IL11RA are involved in crucial processes such as cell proliferation, differentiation, invasion, and metastasis in various cancers, significantly influencing tumorigenesis and progression (80). In lung adenocarcinoma, studies using transcriptomic analysis and Mendelian randomization have revealed that IL11RA expression is downregulated in tumor tissues and exhibits a significant negative correlation with lung adenocarcinoma risk, suggesting a potential tumor suppressor role (81). This study confirmed a negative association between IL11RA expression and bladder cancer risk, consistent with previous findings in lung adenocarcinoma. This causal relationship provides genetic evidence to support the development of targeted therapeutic strategies. Similar findings have been reported in non-small cell lung cancer (NSCLC), where IL11RA also demonstrated tumor-suppressor activity, and its expression level was negatively correlated with NSCLC risk (82). Combined with immune infiltration analysis, IL11RA expression was found to be significantly negatively correlated with the increased infiltration of naive B cells and activated dendritic cells in tumor tissues, suggesting that IL11RA may regulate the bladder tumor immune microenvironment. Based on existing studies and the results of this study, we speculate that IL11RA may influence the development of bladder cancer. Although there are currently no direct clinical interventions targeting IL11RA, its potential value in predicting the efficacy of targeted therapy for bladder cancer warrants further investigation.

This study is the first to report that KCNMB1 (potassium calcium-activated channel subfamily M regulatory beta subunit 1) is significantly downregulated in bladder cancer and is negatively correlated with disease risk, suggesting tumor suppressor gene characteristics. KCNMB1 encodes the β subunit of the large-conductance calcium-activated potassium channel (BK channel), which plays a key role in regulating cellular membrane potential homeostasis, excitability, and contractility by integrating voltage and calcium signals in fundamental physiological processes (83). Notably, BK channels have been shown to regulate synovial cell migration and proliferation (84) and dermal fibroblast proliferation (85), indicating their potential roles in cell proliferation-related diseases. Through multi-omics analysis combined with experimental validation, this study revealed the expression profile of KCNMB1 in bladder cancer tissues and established its association with tumor-associated immune cells for the first time. Furthermore, KCNMB1 expression levels were significantly negatively correlated with the infiltration of naive B cells and activated dendritic cells in tumor tissues, suggesting that this gene may participate in regulating the bladder cancer immune microenvironment. Although research on KCNMB1 in tumors remains limited, the gene expression features and their links to the immune microenvironment discovered in this study provide important clues for further exploration of its potential value as a biomarker for bladder cancer. It should be noted that the molecular mechanisms of KCNMB1 in bladder cancer, particularly the relationship between BK channel function mediated by KCNMB1 and tumor development, require further experimental elucidation.

This study revealed the key role of PDLIM7 (PDZ and LIM domain protein 7) as an immune-related gene in bladder cancer. This gene encodes a non-secretory intracellular protein that contains a PDZ domain and three LIM domains. The PDZ domain interacts with actin-binding proteins, such as β-myosin, and participates in the regulation of cytoskeletal dynamics and signal transduction (86–88). Notably, PDLIM7 may serve as a novel regulator of the p53 pathway by forming a ternary complex with Enigma and MDM2 proteins (89), As the “guardian of the genome,” p53 is a critical tumor suppressor gene playing central roles in cell cycle regulation, DNA damage repair, and apoptosis (90). Studies have indicated that over 50% of tumors exhibit p53 dysfunction, and loss of the p53 pathway is common in human cancers. Thus, targeting the p53 pathway has become a crucial strategy for cancer therapy (91). This study is the first to show that PDLIM7 is significantly downregulated in bladder cancer tissues and that its expression is negatively correlated with disease risk. Previous research has shown that PDLIM7 participates directly or indirectly in the development of various cancers (92), further suggesting that it may exert tumor-suppressive effects through regulation of the p53 signaling pathway. Although this study revealed an important regulatory role of PDLIM7 in bladder cancer, its specific molecular mechanisms and clinical translational potential require systematic validation through *in vitro* and *in vivo* functional experiments.

This study conducted an integrative analysis of multiple databases, with potential heterogeneity effectively controlled through standardized analytical procedures and cross-platform validation (TCGA/GEO validation). Additionally, biological experimental validation was performed using qRT-PCR to ensure reliability of the results. Although immune-related biomarkers for bladder cancer have been identified by integrating multi-source transcriptomic data and GWAS databases, several limitations remain that need to be addressed in future research. Notably, Yan et al. incorporated machine learning algorithms and drug sensitivity analyses in transcriptomic studies, highlighting their significant potential for clinical application through integration with clinical features (93). Liu et al. employed single-cell transcriptomics to precisely dissect tumor microenvironment heterogeneity, thereby providing a new paradigm for immune-related gene research (94). Although this study strengthened the Mendelian randomization validation of GWAS data, further enhancement is needed to integrate multi-omics analytical techniques. Future research will combine machine learning, drug sensitivity prediction, and single-cell transcriptomic technologies to focus on the characteristics of immune cells within the tumor microenvironment and the clinical application potential of the key marker genes identified in this study. As GWAS databases continue to be updated and improved, Mendelian randomization results may change. To explore potential molecular markers associated with bladder cancer pathogenesis, we conducted comprehensive correlation analyses. Nonetheless, further mechanistic studies, including *in vitro* and *in vivo* experiments, are essential to fully elucidate the roles of key genes in bladder cancer development and progression.

## 5 Conclusion

This study used multi-source transcriptomic data combined with Mendelian randomization analyses, including differential gene expression analysis, weighted gene co-expression network analysis (WGCNA), and Mendelian randomization, to identify eight candidate genes that were significantly negatively associated with bladder cancer risk: LIMS2, TP53INP2, IRAK3, STX2, CYP27A1, IL11RA, KCNMB1, and PDLIM7. ROC curve analysis and validation using independent datasets confirmed the strong potential of these eight genes as diagnostic biomarkers for bladder cancer. *In vitro* qRT-PCR validation in bladder cancer cell lines showed downregulation of LIMS2, IRAK3, STX2, IL11RA, KCNMB1, and PDLIM7, consistent with bioinformatics findings, suggesting that these genes may serve as potential therapeutic targets. Through integrated multi-omics analysis and experimental validation, six genes, LIMS2, IRAK3, STX2, IL11RA, KCNMB1, and PDLIM7, were selected as potential immune-related biomarkers for bladder cancer, providing promising biomarkers and therapeutic targets for personalized treatment.

## Data Availability

The original contributions presented in the study are included in the article/**Supplementary Material**. Further inquiries can be directed to the corresponding author/s.
