# Supplementary material for: Exploring of bladder cancer immune-related genes and potential therapeutic targets based on transcriptomic data and Mendelian randomization analysis
Source: Front Immunol. 2025 Jul 18;16:1607098. doi: 10.3389/fimmu.2025.1607098 (PMC12313568; doi:10.3389/fimmu.2025.1607098)
Supplement: Supplementary file 1 [file DataSheet1.docx]

Supplementary Material

# Supplementary Figures

Figure S1: Funnel plot of the eight genes causally associated with bladder cancer.

Figure S2: Scatter plot of the eight genes causally associated with bladder cancer.

Figure S3: Leave-one-out sensitivity analysis of the eight genes causally associated with bladder cancer.

Figure S4. GO and KEGG enrichment analysis of key genes in bladder cancer.

Figure S5. Xcell analysis in bladder cancer.

Figure S6. Box plots showing differential expression analysis of key genes in TCGA and GSE7476 datasets.

Figure S7: Box plots showing the differential analysis of qRT-PCR experiments for the eight key genes.

## Supplementary Figures


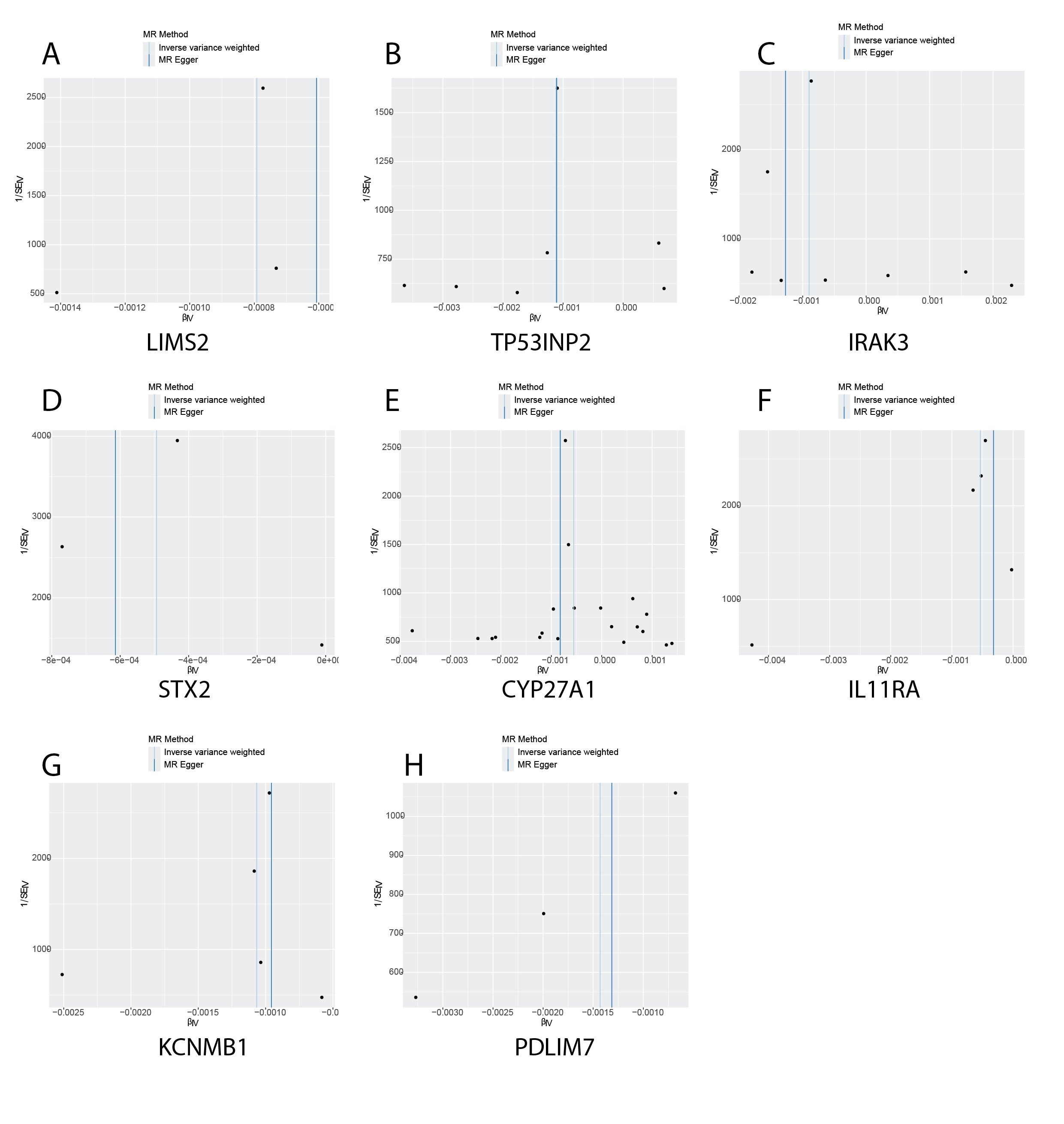


**Supplementary Figure S1.** (A-H) MR effect size of key genes on bladder cancer; (A) LIMS2; (B) TP53INP2; (C) IRAK3; (D) STX2; (E) CYP27A1; (F) IL11RA; (G)KCNMB1; (H) PDLIM7.


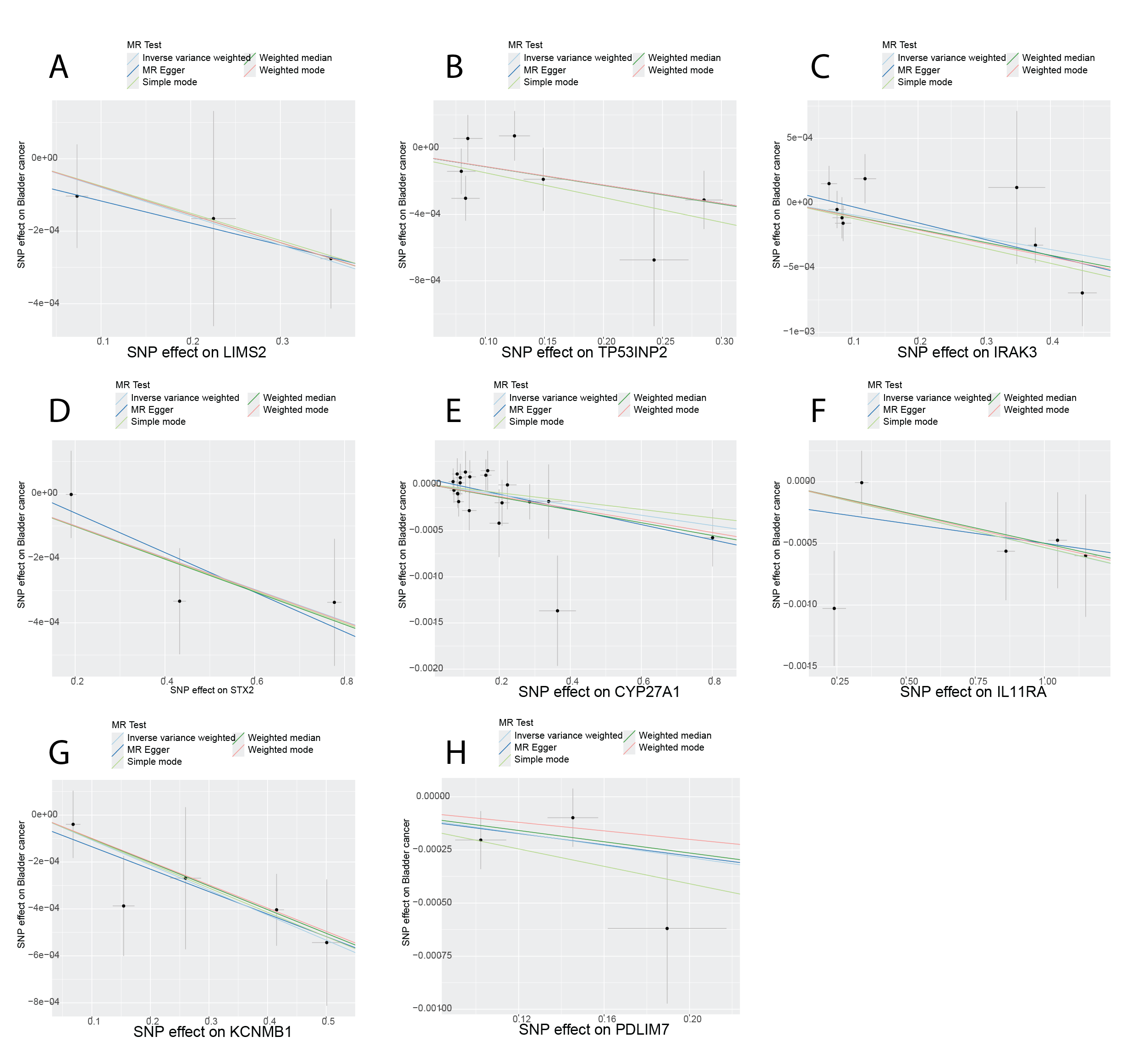


**Supplementary Figure S2.** Scatter plot of eight genes causally linked to bladder cancer. (A-H) MR effect size of key genes on bladder cancer; (A) LIMS2; (B) TP53INP2; (C) IRAK3; (D) STX2; (E) CYP27A1; (F) IL11RA; (G) KCNMB1; (H) PDLIM7


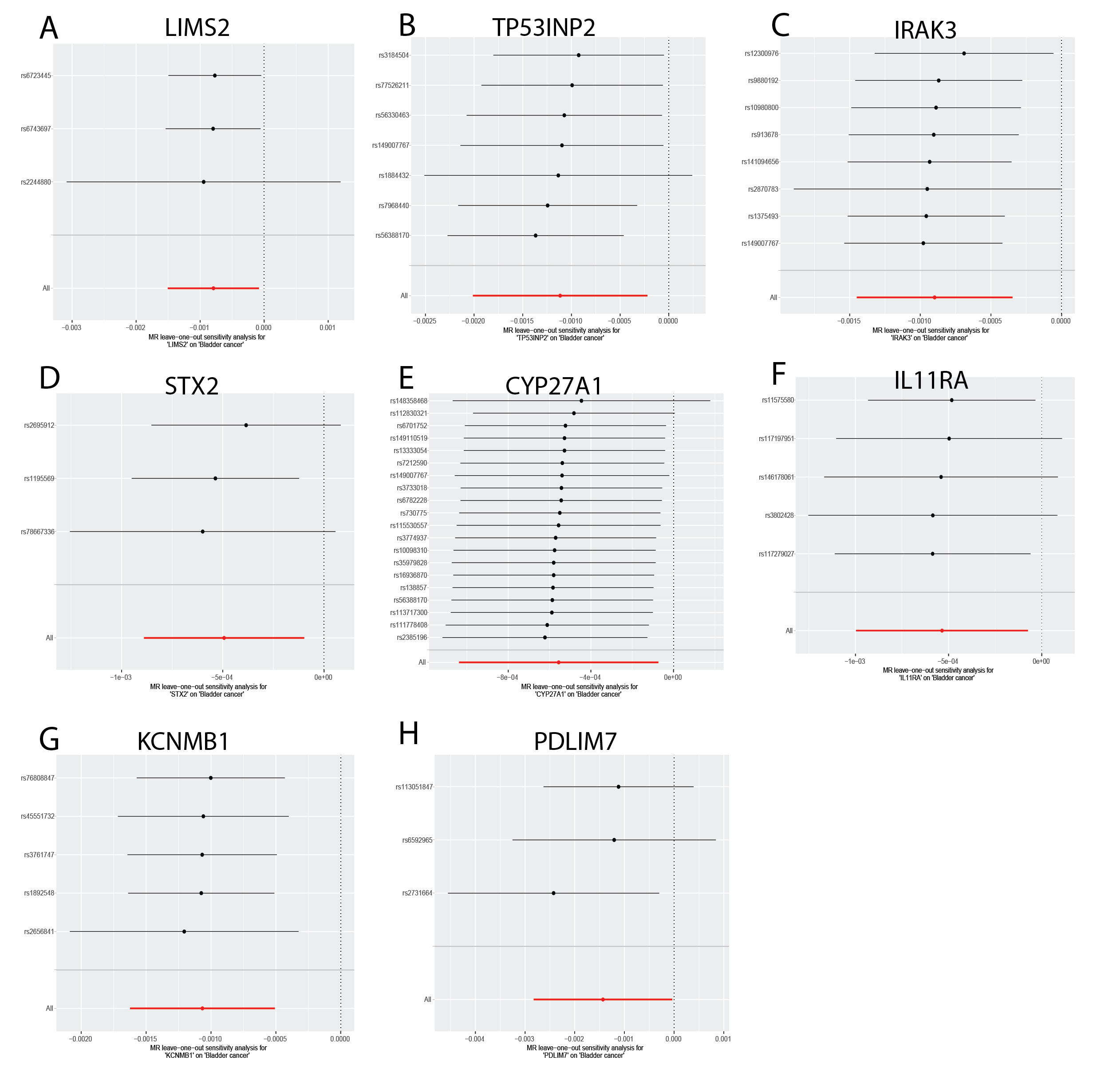


**Supplementary Figure S3.** Leave-one-out analysis plot of eight genes causally linked to bladder cancer. (A-H) MR effect size of key genes on bladder cancer; (A) LIMS2; (B) TP53INP2; (C) IRAK3; (D) STX2; (E) CYP27A1; (F) IL11RA; (G) KCNMB1; (H) PDLIM7


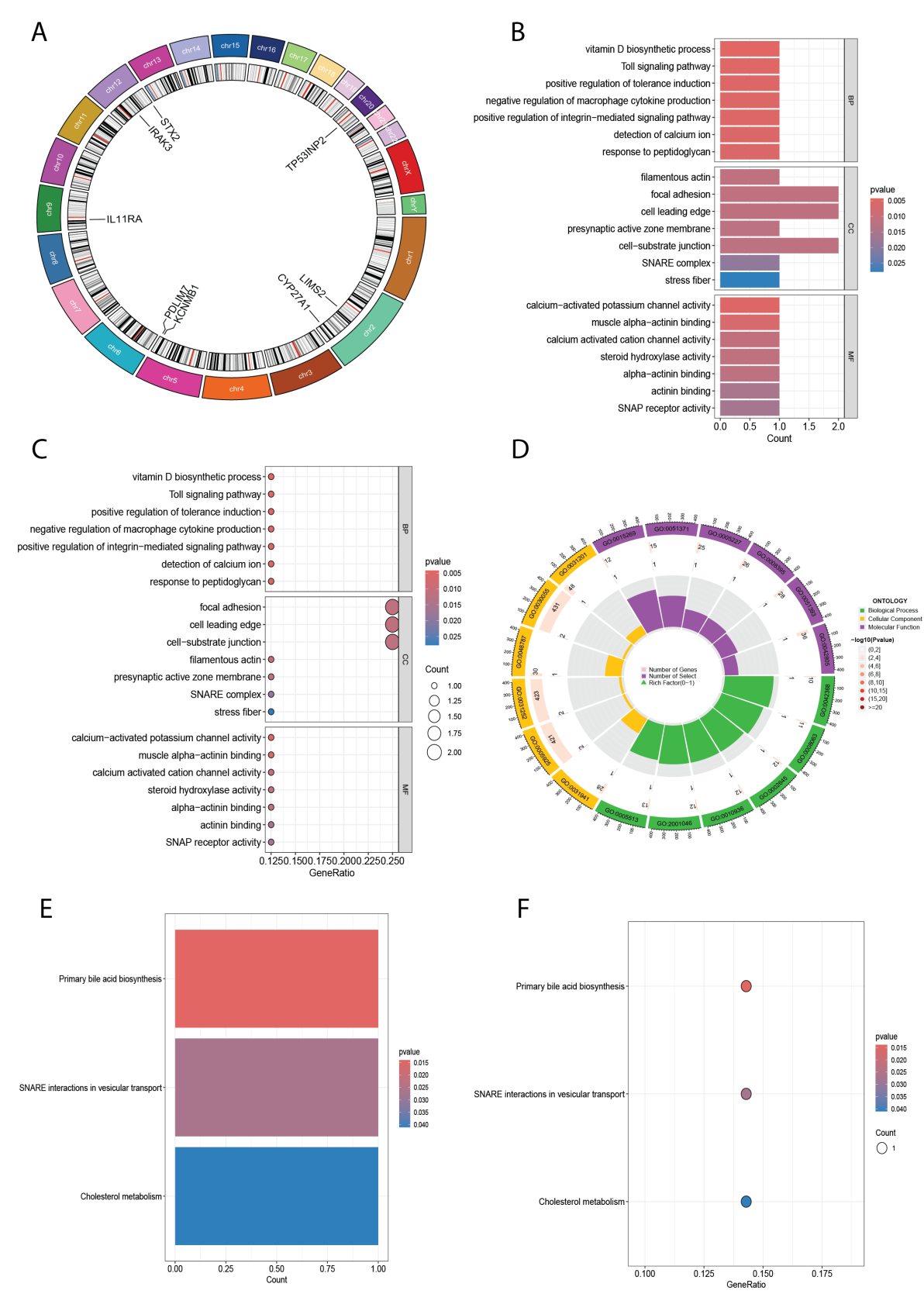


**Supplementary Figure S4.** GO and KEGG enrichment analysis of key genes in bladder cancer. (A) Circos plot of co-expressed genes; (B) Bar plot of GO enrichment analysis for the eight key genes; (C) Bubble plot of GO enrichment analysis for the eight key genes; (D) Bar plot of KEGG enrichment analysis for the eight key genes; (E) Bubble plot of KEGG enrichment analysis for the eight key genes.(The Circos plot depicts a circular chromosome map. Different colored regions represent different chromosomes, with the locations of eight co-expressed genes marked.The size of the bubble indicates the strength of the P value.)


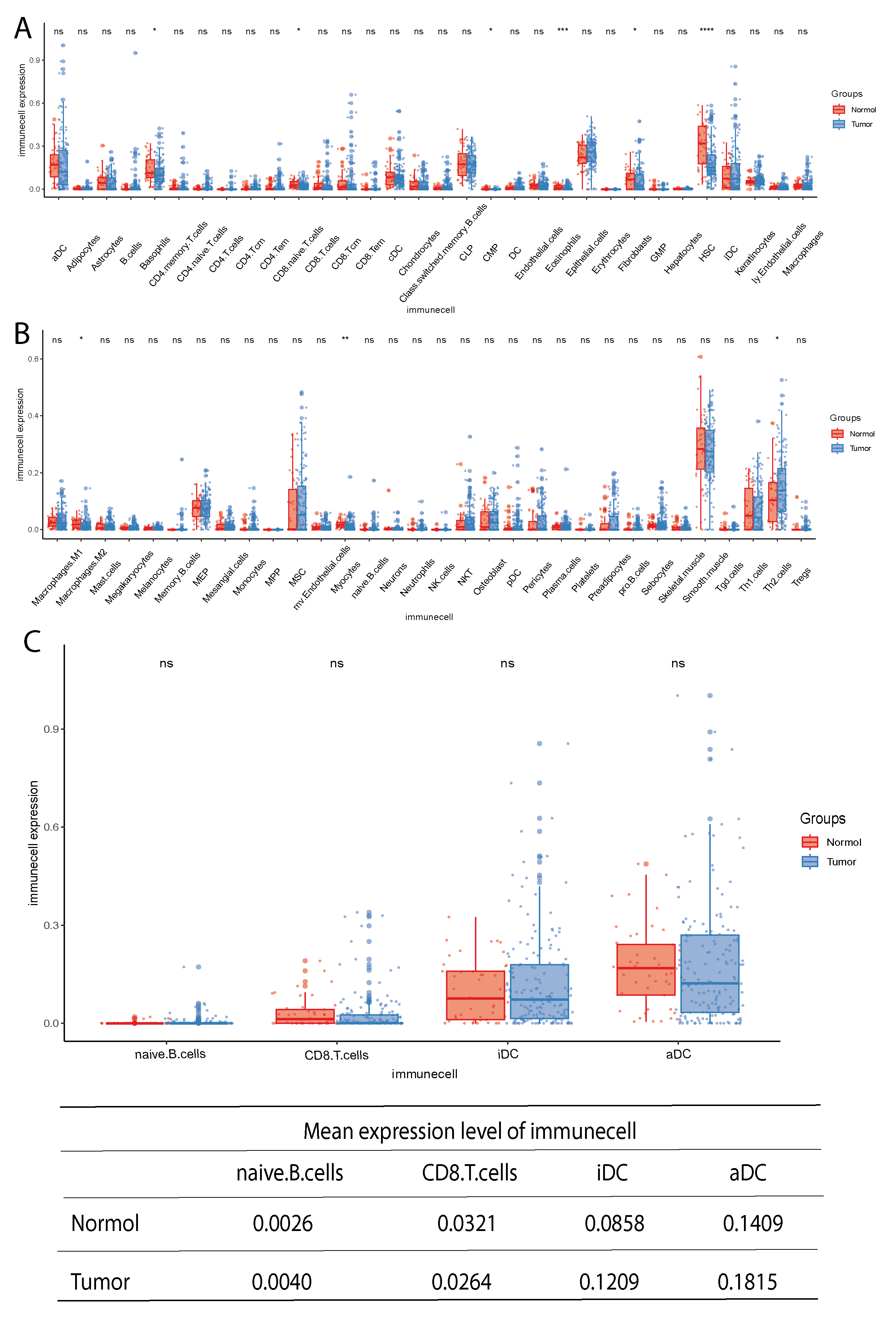


**Supplementary Figure S5.** Xcell analysis in bladder cancer. (A-B) Box plots demonstrate differential infiltration landscapes of 64 immune cell types between bladder cancer and normal tissues. (C) Box plots illustrate distinct infiltration patterns of 4 immune cell subsets in bladder cancer versus normal samples.


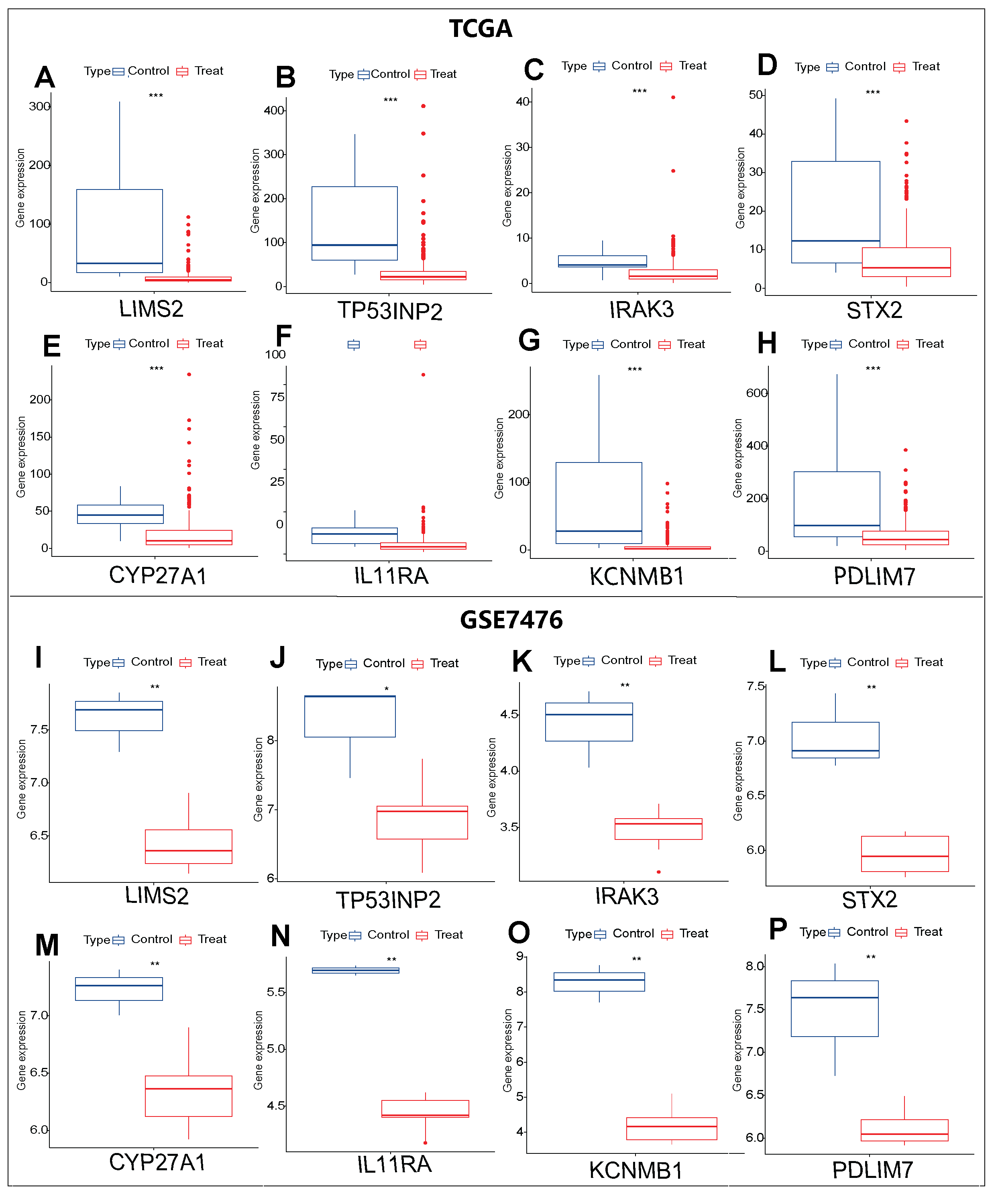


**Supplementary Figure S6.** Box plots showing differential expression analysis of key genes in TCGA and GSE7476 datasets. (A-H) Box plots showing the differential expression analysis results of LIMS2, TP53INP2, IRAK3, STX2, CYP27A1, IL11RA, KCNMB1, and PDLIM7 in the TCGA dataset; (I-P) Box plots showing the differential expression analysis results of LIMS2, TP53INP2, IRAK3, STX2, CYP27A1, IL11RA, KCNMB1, and PDLIM7 in the GSE7476 dataset. Differences between groups were assessed using the Wilcoxon rank-sum test. Each p-value is shown above the corresponding box plot (NS: p > 0.05; *: p ≤ 0.05; **: p ≤ 0.01; ***: p ≤ 0.001; ****: p ≤ 0.0001).


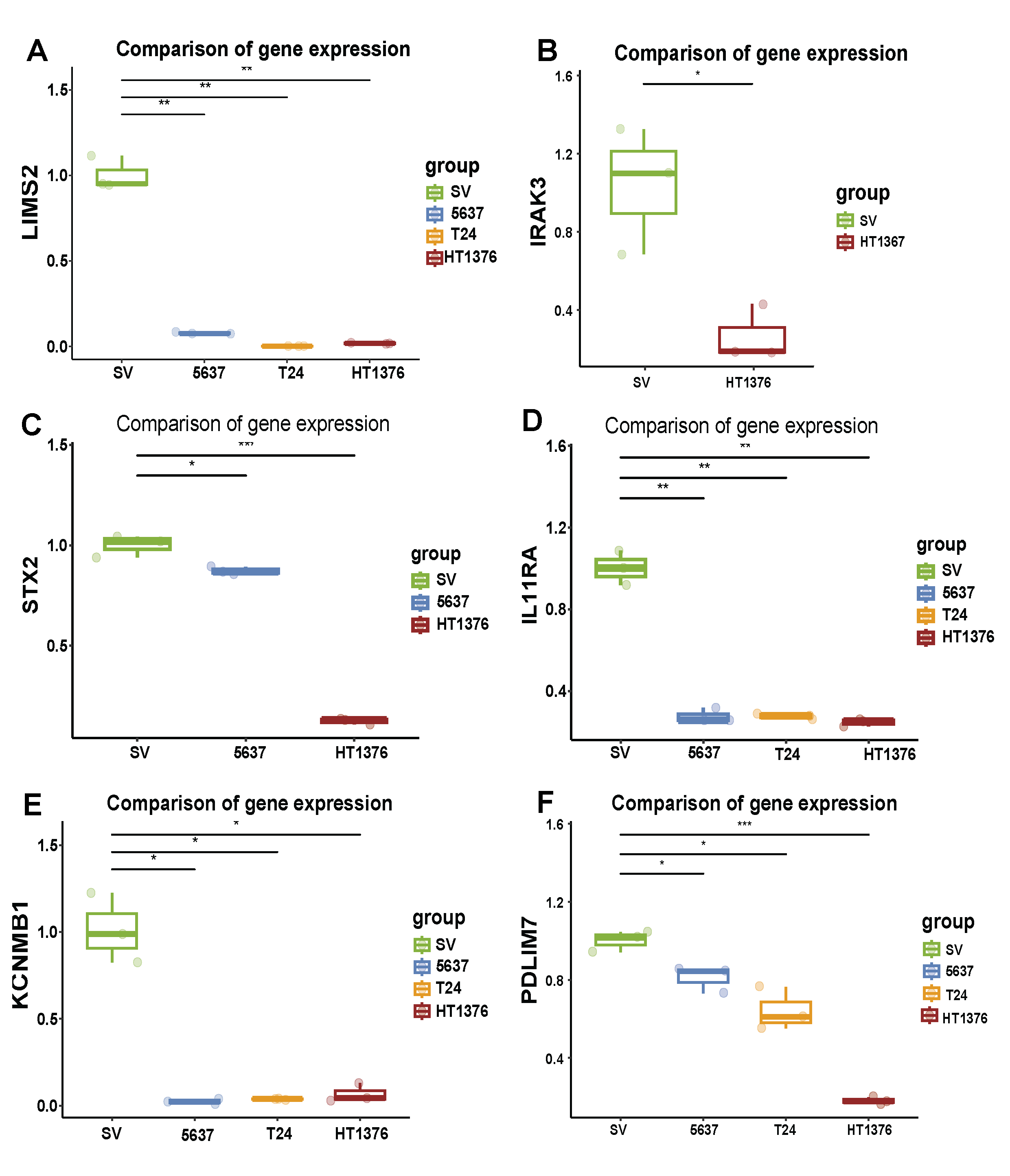


**Supplementary Figure S7.** Real-time quantitative PCR (qPCR) and differential analysis box plots of key genes. (A-F) Box plots showing differential expression analysis of eight key genes in bladder normal cells (SV) and bladder cancer cell lines (5637, T24, and HT1376): (A) LIMS2; (B) IRAK3; (C) STX2; (D) IL11RA; (E) KCNMB1; (F) PDLIM7. (NS: p > 0.05; *: p ≤ 0.05; **: p ≤ 0.01; ***: p ≤ 0.001; ****: p ≤ 0.0001).
